# Direct Capsid Labeling of Infectious HIV-1 by Genetic Code Expansion Allows Detection of Largely Complete Nuclear Capsids and Suggests Nuclear Entry of HIV-1 Complexes via Common Routes

**DOI:** 10.1128/mbio.01959-22

**Published:** 2022-08-16

**Authors:** Sandra Schifferdecker, Vojtech Zila, Thorsten G. Müller, Volkan Sakin, Maria Anders-Össwein, Vibor Laketa, Hans-Georg Kräusslich, Barbara Müller

**Affiliations:** a Department of Infectious Diseases, Virology, University Hospital Heidelberg, Heidelberg, Germany; b German Center for Infection Research, partner site Heidelberg, Germany; Columbia University Medical Center

**Keywords:** HIV-1, capsid, click labeling, amber suppression, genetic code expansion, primary CD4+ T cells, electron microscopy, correlative microscopy, STED, superresolution microscopy, human immunodeficiency virus

## Abstract

The cone-shaped mature HIV-1 capsid is the main orchestrator of early viral replication. After cytosolic entry, it transports the viral replication complex along microtubules toward the nucleus. While it was initially believed that the reverse transcribed genome is released from the capsid in the cytosol, recent observations indicate that a high amount of capsid protein (CA) remains associated with subviral complexes during import through the nuclear pore complex (NPC). Observation of postentry events via microscopic detection of HIV-1 CA is challenging, since epitope shielding limits immunodetection and the genetic fragility of CA hampers direct labeling approaches. Here, we present a minimally invasive strategy based on genetic code expansion and click chemistry that allows for site-directed fluorescent labeling of HIV-1 CA, while retaining virus morphology and infectivity. Thereby, we could directly visualize virions and subviral complexes using advanced microscopy, including nanoscopy and correlative imaging. Quantification of signal intensities of subviral complexes revealed an amount of CA associated with nuclear complexes in HeLa-derived cells and primary T cells consistent with a complete capsid and showed that treatment with the small molecule inhibitor PF74 did not result in capsid dissociation from nuclear complexes. Cone-shaped objects detected in the nucleus by electron tomography were clearly identified as capsid-derived structures by correlative microscopy. High-resolution imaging revealed dose-dependent clustering of nuclear capsids, suggesting that incoming particles may follow common entry routes.

## INTRODUCTION

The cone-shaped capsid that encases the viral RNA genome and replication proteins is a characteristic feature of infectious human immunodeficiency virus type 1 (HIV-1) particles. Data obtained by many research groups over the past decade have revised our understanding of the role of the mature capsid in HIV-1 replication, placing this structure at the center stage of postentry replication steps (reviewed in references 1–4). Upon fusion of the virion envelope with the cell membrane, the capsid, which consists of ~1,200 to 1,500 monomers of the capsid protein CA (5), is released into the cytosol. It then usurps host cell factors to traffic toward the nucleus. Reverse transcription of the viral RNA into double-stranded DNA (dsDNA) is initiated during passage of the subviral structure through the cytosol. Following import into the nucleus, the viral dsDNA is covalently integrated into the host cell genome by the viral integrase (IN). Prior to integration, the surrounding capsid shell needs to release the dsDNA in a process termed uncoating. The precise mechanisms, location, and timing of HIV-1 capsid uncoating are still under investigation.

Initially, the HIV-1 capsid was presumed to rapidly dissociate upon cell entry, based on little or no CA detected associated with isolated postentry complexes (reviewed in reference 6). Rapid or gradual disassembly in the cytosol was also supported by several studies that applied fluorescence imaging to analyze subviral complexes in infected cells (e.g., references 7–9). However, HIV-1 CA or the capsid lattice was found to directly engage in interactions with various host factors involved in postentry replication steps. Among these capsid-interacting host factors are not only cytosolic proteins, including proteins involved in microtubular transport, but also nucleoporins and even the nuclear protein cleavage and polyadenylation specific factor 6 (CPSF6) (reviewed in references 2, 10, and 11). These findings implied involvement of at least a partial lattice structure in later stages of postentry replication. Furthermore, increasing evidence from imaging-based analyses argued for capsid uncoating at or close to the nuclear pore (12–15) or even indicated passage of (nearly) intact capsids through nuclear pores (16–19). The recent detection of cone-shaped objects in the nuclear pore channel and inside the nucleus by correlative light and electron microscopy (CLEM) (16) and intranuclear separation of CA or IN from reverse transcribed dsDNA (17) also supported the model that the nucleus is the site of HIV-1 uncoating (20).

One explanation for apparent discrepancies between different studies is the methods that have been used for CA detection in fluorescence microscopy. Since the modification of CA by genetic labeling strategies proved to be challenging, most studies applied immunofluorescence (IF) staining or indirect labeling through a capsid binding protein (e.g., references 8, 13, 15, 21–23). A limitation of IF is that staining efficiency may vary substantially depending on the antibody and detection conditions used, as well as on differential exposure or shielding of epitopes due to conformational changes or different intracellular environments. We could indeed show previously that immunostaining efficiency of CA in the nucleus of host cells strongly depends on cell type and experimental conditions (17). Furthermore, IF is incompatible with live cell analyses. Infectious HIV-1 derivatives carrying fluorescent CA would resolve these limitations and allow the direct observation of entering capsids with quantitative analyses.

Direct genetic labeling of viral capsid proteins is challenging, however. Capsid proteins are generally small proteins that need to assemble into ordered multimeric lattices. The resulting assemblies must be stable during virus formation and transmission to a new target cell, but also ready to disassemble in the newly infected cell, requiring structural flexibility of the protomers. Beyond protein-protein interactions involved in capsid assembly itself, capsid proteins generally undergo crucial interactions with other components of the virion, e.g., the viral genome. Finally, the capsid surface represents an essential contact interface between virus and host cell in the early phase of infection, mediating cell entry in the case of nonenveloped viruses or interacting with critical host cell dependency or restriction factors in the case of enveloped viruses. Consequently, a large proportion of the surface exposed amino acids of a viral capsid protein is involved in intermolecular contacts that are crucial for virus replication, which renders these proteins highly susceptible to genetic modification. Fusion of a capsid protein to a relatively large genetic label, e.g., green fluorescent protein (GFP) or other fluorescent proteins, is thus generally prone to severely affect virus infectivity.

These considerations also apply to HIV-1 CA. The protein is encoded as a subdomain of the structural polyprotein Gag, from which it is released by the viral protease (PR) concomitant with virus budding to allow for formation of the mature capsid. With a molecular mass of ~24 kDa, mature CA is of a similar size as GFP. CA hexamers are the core structural elements of the immature Gag shell forming the nascent virus bud in HIV-1-producing cells. Hexamers with a different conformation, together with 12 CA pentamers that allow for bending and curvature, are the building blocks of the mature capsid. CA pentamers and immature and mature hexamers employ different protein-protein interfaces; together, these interfaces involve most of the exposed surface of the CA monomer (reviewed in reference 24). Accordingly, scanning mutagenesis analyses found HIV-1 CA to be highly genetically fragile (25, 26), with up to 89% of single amino acid exchanges tested abolishing or severely affecting virus replication (26). It is thus not surprising that the introduction of genetically encoded labels, GFP or even a small peptide tag, at various positions within HIV-1 CA has resulted in loss or severe reduction of infectivity. Complementation with wild-type (wt) virus, from at least equimolar amounts of wt CA to a substantial molar excess, was essential to restore virus infectivity (18, 27–29). While the use of wt-complemented particles can be sufficient for fluorescent labeling, it is unclear whether the modified CA molecules are an integral part of the mature CA lattice. Only approximately half of the CA molecules present inside the virion are eventually used to form the mature capsid (30, 31), and CA fusion proteins may be preferentially excluded or less stably integrated into the mature lattice.

**FIG 1:**
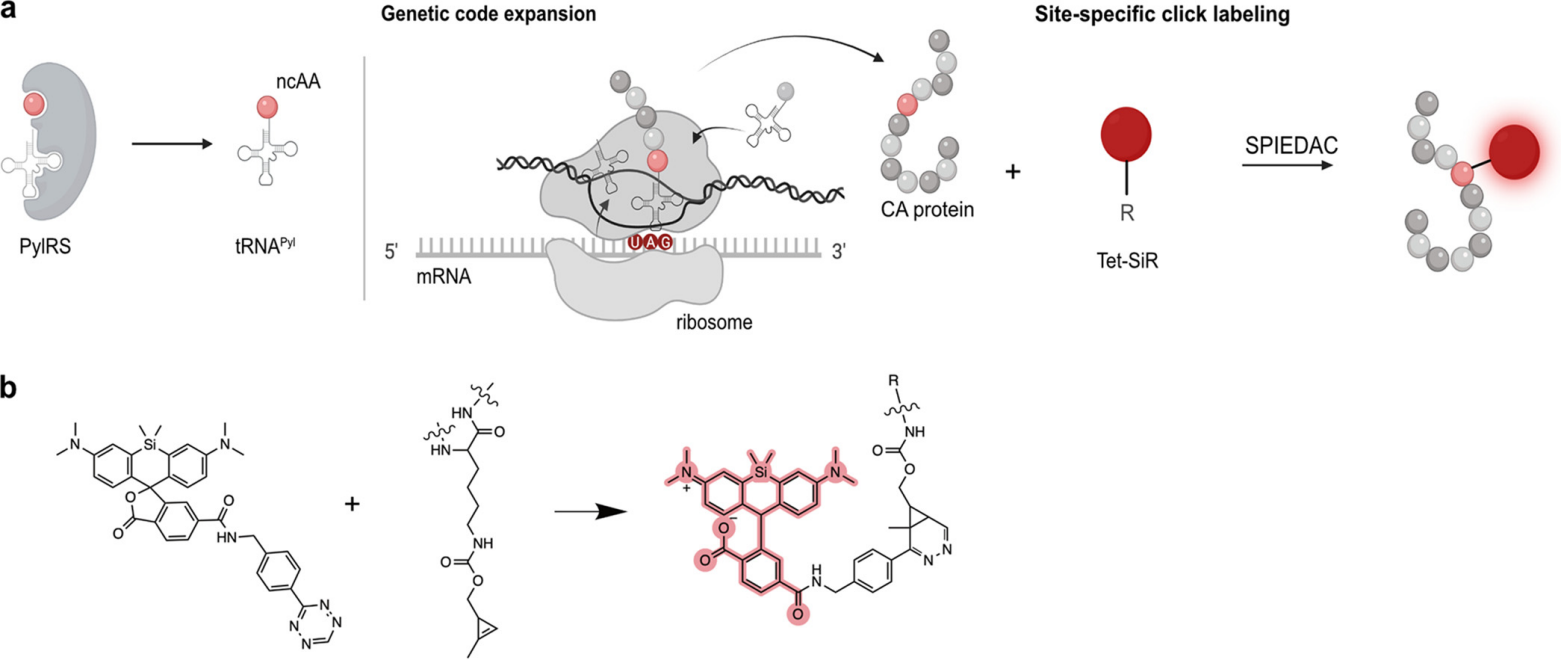
GCE and click labeling of HIV-1 CA. (a) Experimental scheme for GCE and click-labeling. The system used here requires the introduction of an amber stop codon (UAG) at a specific site into the CA coding sequence. A genetically engineered bio-orthogonal tRNA/aminoacyl-tRNA synthetase (PylRS) pair mediates incorporation of a noncanonical amino acid (ncAA) at the chosen position. In a second step, a highly reactive group of the ncAA is covalently linked to a fluorophore carrying a cognate reactive group (e.g., a tetrazine group reacting with a cyclopropene group at the ncAA via strain-promoted inverse electron-demand Diels-Alder cycloaddition [SPIEDAC]). Image created with BioRender.com. (b) The tetrazine-derivative of silicon rhodamine (Tet-SiR) reacts with the strained alkene of the ncAA cyclopropene-l-lysine (CpK) via SPIEDAC. The open SiR conformation results in fluorescence (highlighted in red).

We therefore established and applied a minimal invasive labeling strategy for HIV-1 CA based on genetic code expansion (GCE) and click labeling. This method involves the exchange of a selected amino acid residue in the protein of interest with a noncanonical amino acid (ncAA) carrying a highly reactive bio-orthogonal functional group by a process termed amber suppression (Fig. 1a). This residue is subsequently covalently coupled to a fluorophore functionalized with a cognate reaction partner (Fig. 1a and b; reviewed in, e.g., references 32–34). Using this approach, we generated a CA-labeled HIV-1 derivative that largely retained infectivity. In contrast to previous approaches for direct CA labeling, our minimally modified derivative did not require complementation with wt virus. Direct labeling with a bright and photostable chemical dye allows the application of various imaging methods, i.e., live-cell imaging, superresolution nanoscopy, or CLEM. The click-labeled virus variant thus enabled us to directly assess the amount of CA associated with entering subviral complexes outside and within the nucleus of infected HeLa-derived cells and primary CD4^+^ T cells, to visualize CA-containing structures in the nucleus by nanoscopy and correlative microscopy and to study the effect of the CA-binding drug PF74 on the nuclear complexes.

## RESULTS

### Generation of an HIV-1 variant carrying a bio-orthogonal amino acid within CA.

To allow for minimally invasive labeling of HIV-1 CA by GCE (Fig. 1), we introduced an amber stop codon at a position of interest into the CA coding sequence within the *gag* open reading frame of the proviral plasmid pNLC4-3 (35). In order to avoid GCE modification of the accessory viral protein R (Vpr), which is incorporated into the virion in high amounts (36), we first exchanged the amber stop codon of *vpr* to an opal codon (TGA), resulting in plasmid pNLC4-3*. Although this mutation did not alter the coding sequence of viral proteins or virion infectivity (Fig. S1), the corresponding virus was termed HIV-1* to indicate the modification. Since neither the efficiency of amber suppression in a given sequence context in eukaryotic cells nor the effect of ncAA incorporation on viral functionality can be predicted with certainty, we tested a panel of 18 amber mutations at sites located toward the outer surface of the capsid lattice for suppression efficiency and virus infectivity (S. Schifferdecker, V. Sakin, et al., unpublished data). Based on a comparison of Gag expression levels and viral infectivity upon ncAA incorporation, we selected a virus variant in which residue alanine 14 in CA was replaced by a noncanonical amino acid (HIV-1*CA14^ncAA^) for further analyses.

10.1128/mbio.01959-22.1 FIG S1 Characterization of HIV-1*. The proviral plasmid pNLC4-3 was modified in order to allow for site-specific click labeling of the viral CA. The amber stop codon of the *vpr* open reading frame was mutated into an opal TGA stop codon to avoid GCE modification of Vpr. HEK293T cells were transfected with pNLC4-3 or pNLC4-3* and pNESPylRS-eRF1dn-tRNA and grown in the presence of 500 μM CpK. At 48 h p.t., supernatant of transfected cells was harvested, filtered, and concentrated via ultracentrifugation through a sucrose cushion. (a) Quantification of RT activity determined in an SG-PERT assay. (b) Relative infectivity of HIV-1* measured *via* luciferase assay in TZM-bl reporter cells. Infectivity was normalized to HIV-1_NL4-3_ infectivity measured in parallel. Graphs show mean values and SD of five replicates performed in three independent experiments. Download FIG S1, TIF file, 0.1 MB. Copyright © 2022 Schifferdecker et al. 2022 Schifferdecker et al. https://creativecommons.org/licenses/by/4.0/ This content is distributed under the terms of the Creative Commons Attribution 4.0 International license.

**FIG 2:**
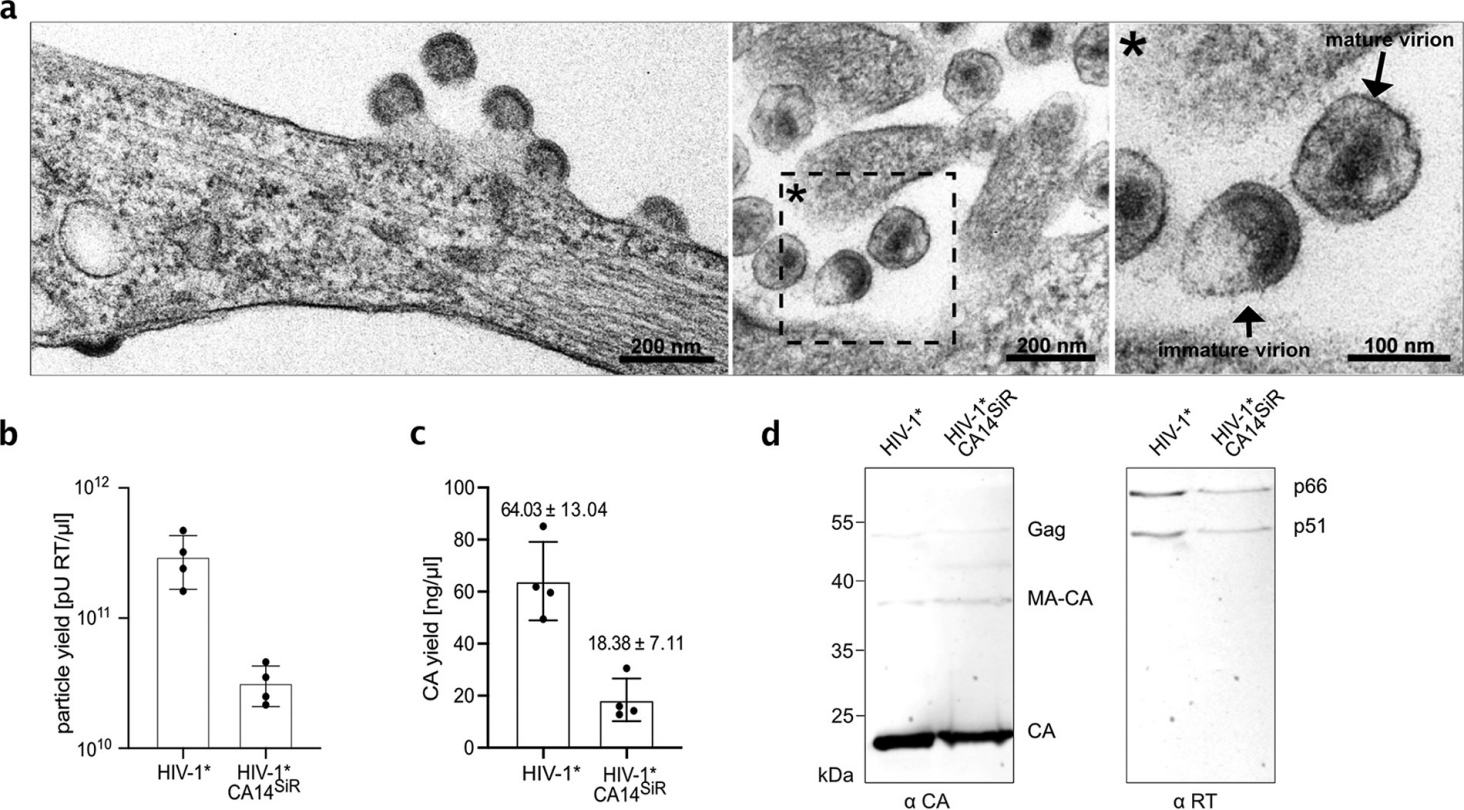
Production and characterization of click-labeled HIV-1 (HIV-1*CA14^SiR^). (a) Morphology of HIV-1*CA14^ncAA^ assembly sites and particles. HEK293T cells were cotransfected with pNLC4-3*CA14^TAG^ and pNESPylRS-eRF1dn-tRNA and grown in the presence of 500 μM CpK. At 44 h p.t., cells were fixed, embedded, and analyzed by thin-section EM as described in Materials and Methods. (b and c) Virus production. Click-labeled particles were prepared from the supernatant of HEK293T cells cotransfected with either pNLC4-3* or pNLC4-3*CA14^TAG^ and pNESPylRS-eRF1dn-tRNA and grown in the presence of 500 μM CpK as described in Materials and Methods. Particle yield in the final preparations was determined via quantitation of RT activity (SG-PERT assay [79]) (b) and by determination of CA amounts using quantitative immunoblot as described in Materials and Methods (c). The graphs show mean values and SD from four independent experiments. (d) Immunoblot analysis of virus preparations. Five microliters of HIV-1* and HIV-1*CA14^SiR^ particle lysates was separated by SDS-PAGE, and proteins were transferred to nitrocellulose membranes by semidry blotting. Viral proteins were detected using the indicated polyclonal antisera. Bound antibodies were detected by quantitative immunofluorescence with a Li-COR CLx infrared scanner, using secondary antibodies and protocols according to the manufacturer’s instructions. Positions corresponding to Gag/Gag-Pol and processing products are indicated at right.

For virus preparation, HEK293T cells were cotransfected with the respective mutant proviral plasmid and pNESPylRS-eRF1dn-tRNA. The latter plasmid encodes for a complete amber suppression system, consisting of modified tRNA, a cognate genetically engineered pyrrolysine aminoacyl-tRNA synthetase (37), and a dominant negative version of the eukaryotic release factor eRF1 that improves amber suppression efficiency in eukaryotic cells (38). To produce functionalized virus particles, cells were grown in the presence of the small ncAA cyclopropene lysine (CpK). While truncation of Gag at position 14 of CA would prevent virus formation, incorporation of CpK by amber suppression should result in the expression of full-length Gag and thereby promote HIV-1 particle assembly. Immunoblot analysis of cell lysates indeed demonstrated the presence of full-length Gag polyprotein precursor when HIV-1*CA14^TAG^-expressing cells were grown in the presence of CpK, whereas full-length Gag was not detected when CpK was omitted from the growth medium (Fig. S2a). Thin-section electron microscopy (EM) revealed late budding sites and immature-like as well as mature-like virions at the plasma membrane and in the vicinity of HIV-1*CA14^TAG^-expressing cells that were morphologically indistinguishable from typical HIV-1 wild-type (wt) budding sites and virions (Fig. 2a). Virus release efficiency from transfected cells, as estimated by immunoblot analysis of cell and particle lysates, was comparable for wt and HIV-1*CA14^CpK^ (Fig. S2b). We concluded that Gag expression of HIV-1*CA14^TAG^ is ncAA dependent and the modified CA domain is competent for immature and mature lattice assembly.

10.1128/mbio.01959-22.2 FIG S2 Detection of full-length Gag Pr55 in the presence or absence of CpK. Immunoblot analysis of cell lysate of transfected cells. HEK293T cells were cotransfected with pNLC4-3* or pNLC4-3*CA14^TAG^ and pNESPylRS-eRF1dn-tRNA and grown in the presence or absence of 500 μM CpK. (a) Cell lysates were separated by SDS-PAGE and proteins were transferred to a nitrocellulose membrane by semidry blotting. Gag-derived proteins were detected using polyclonal rabbit antiserum raised against recombinant HIV-1 MA. Note that the truncated MA-CA14 protein (~16.3 kDa) produced in the absence of amber suppression by HIV-1*CA14^TAG^ is not resolved from mature wt MA (~14.8 kDa). Bound antibody was detected using a Li-COR CLx infrared scanner, employing secondary antibody and protocols according to the manufacturer's instructions. (b) Supernatant of pNLC4-3* or pNLC4-3*CA14^TAG^ and pNESPylRS-eRF1dn-tRNA transfected HEK293T cells, grown in a 6-well plate and in the presence of 500 μM CpK, was harvested at 48 h p.t., filtered, and concentrated *via* ultracentrifugation through a 20% (wt/wt) sucrose cushion. Cell and particle lysates were separated by SDS-PAGE and proteins were transferred to a nitrocellulose membrane by semidry blotting. Gag-derived proteins were detected by quantitative immunoblot (Li-Cor) using a polyclonal rabbit antiserum raised against HIV-1 CA and purified recombinant CA protein as a standard. The ratio of CA amounts detected in the particle lysate to total amounts of Gag-derived proteins in cell and particle lysates was calculated to estimate release efficiency for pNLC4-3* (11.6%) and pNLC4-3*CA14^TAG^ (10.9%) transfected cells. Bar graphs represent mean and SD of three technical replicates. Download FIG S2, TIF file, 0.2 MB. Copyright © 2022 Schifferdecker et al. 2022 Schifferdecker et al. https://creativecommons.org/licenses/by/4.0/ This content is distributed under the terms of the Creative Commons Attribution 4.0 International license.

### Characterization of click-labeled HIV-1 virions.

We next prepared virus particles from the supernatant of HIV-1*CA14^ncAA^-producing cells and subjected them to click labeling using the membrane-permeable dye silicon rhodamine tetrazine (SiR-Tet [39]; Fig. 1b), generating HIV-1*CA14^SiR^. As a control, HIV-1* wt particles were prepared under amber suppression conditions and stained in parallel. Consistent with the detection of viral assembly sites and particles in electron micrographs of transfected cells (Fig. 2a), virus was recovered from the tissue culture supernatant of HIV-1*CA14^ncAA^-expressing cells. Particle yields were moderately reduced compared to the HIV-1* wt control, in line with the fact that amber suppression is usually incomplete in eukaryotic cells (optimal ncAA incorporation efficiencies in the range of ~25 to 50%, e.g., references 38, 40). On average, we obtained 5- to 10-fold lower yields for HIV-1*CA14^SiR^ compared to HIV-1* as determined by reverse transcription (RT) activity (Fig. 2b) and CA content (Fig. 2c) of particle preparations. Comparison of stained and unstained preparations demonstrated that particle yield, assessed by RT activity of virus preparations, was not affected by the click-labeling procedure (Fig. S3). Consistent with the observation of morphologically mature particles by EM, click-labeled particles displayed regular Gag and GagPol processing products (Fig. 2d), with clear bands for mature CA (p24) and mature RT heterodimer (p51, p66).

10.1128/mbio.01959-22.3 FIG S3 Effect of click-labeling on particle yield. HEK293T cells seeded in 6-well plates were transfected with and pNLC4-3* or pNLC4-3*CA14^TAG^ and grown in the presence of 500 μM CpK. Tissue culture supernatant was harvested at 48 h p.i. One-half of the supernatant was incubated with 250 nM SiR-Tet for 30 min, and the other half was left untreated. Subsequently, particles were pelleted by ultracentrifugation through a 20% (wt/wt) sucrose cushion, and pellets were resuspended in 30 μL PBS. The RT activity of unstained (black) and stained (red) samples was determined by SG-PERT. The graph shows data from four parallel transfections; lines connect samples prepared from the same tissue culture supernatant. Download FIG S3, TIF file, 0.10 MB. Copyright © 2022 Schifferdecker et al. 2022 Schifferdecker et al. https://creativecommons.org/licenses/by/4.0/ This content is distributed under the terms of the Creative Commons Attribution 4.0 International license.

### Fluorescence labeling and infectivity of click-labeled virions.

**FIG 3:**
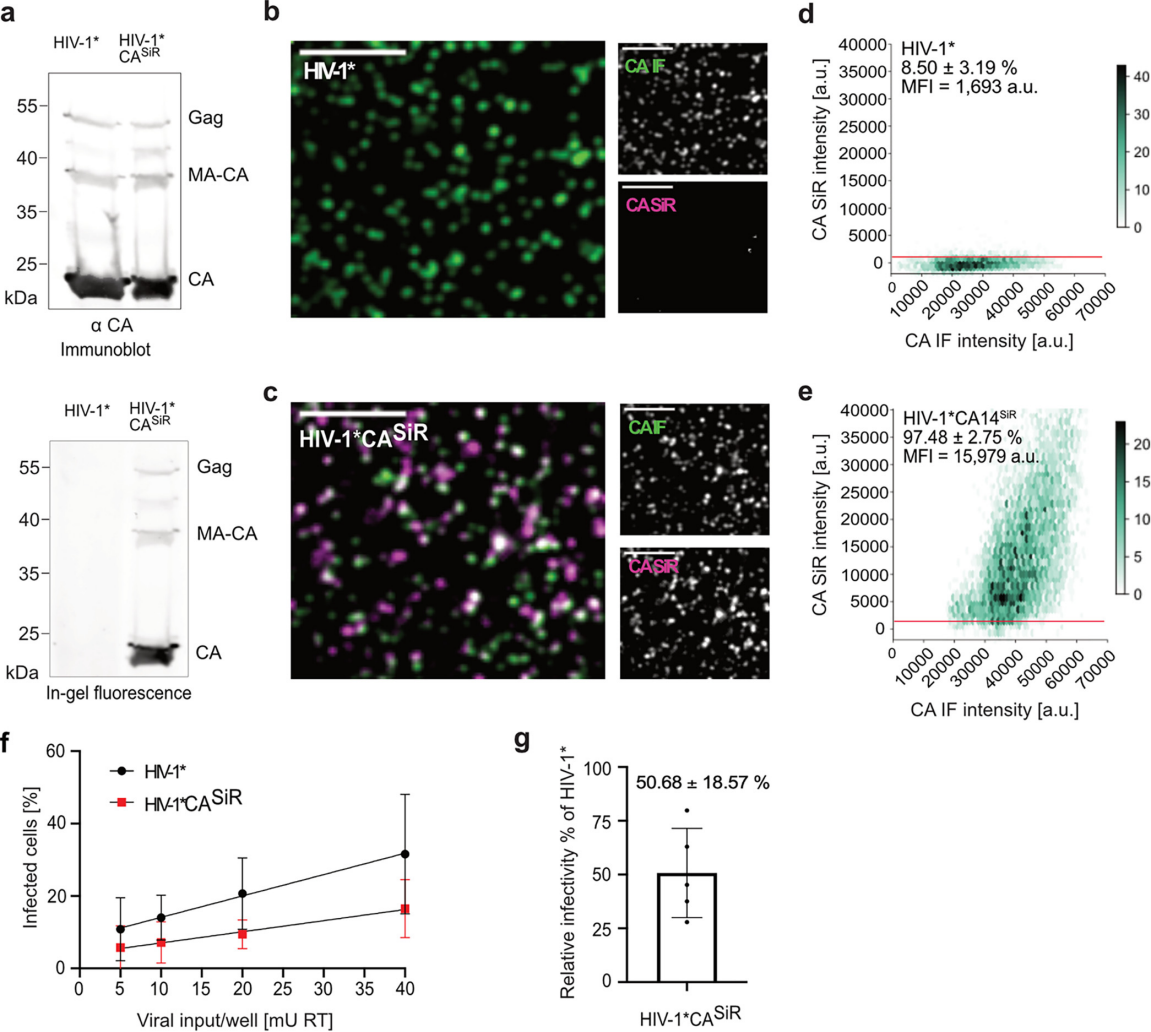
Characterization of CA click-labeled particles. (a) Specific fluorescent labeling of CA14^CpK^. Immunoblot analysis of virus preparations. Twenty microliters of HIV-1* and HIV-1*CA14^SiR^ particle lysates was separated by SDS-PAGE. In-gel fluorescence (bottom) was detected by scanning the gel on a Li-COR Clx infrared scanner set at an emission wavelength of 700 nm. Subsequently immunoblot analysis (top) was performed using the previously scanned gel. Proteins were transferred from the gel to a nitrocellulose membrane by semidry blotting. Viral proteins were detected using polyclonal antiserum raised against recombinant CA. Bound antibodies were detected by quantitative immunofluorescence with a Li-COR CLx infrared scanner, using secondary antibody and protocol according to the manufacturer's instructions. Please note that at the high amounts of particles loaded for efficient in-gel fluorescence detection the immunoblot detection is not within the linear range of the method, so that contaminating precursor bands appear overrepresented. (b to e) Analysis of labeling efficiency. Particles harvested from the supernatant of virus-producing HEK239T cells were subjected to click labeling. Particles were then immobilized on PEI coated chamber slides, fixed, and permeabilized. (b and c) Particles were immunostained using antiserum raised against HIV-1 CA, and specimens were imaged by SDCM. Scale bars = 5 μm. (d and e) Hexabin plots of particles detected in panels b and c. Mean intensities of CA(SiR) are plotted against mean intensity CA(IF) for HIV-1* and HIV-1*CA14^SiR^. The color intensity of the hexagons corresponds to the number of particles displaying the indicated intensity values. The graphs represent pooled data from 12 fields of view from three independent virus preparations. The red line indicates the threshold *t* = 1,000. (f) Infectivity of click-labeled particles. The indicated virus particles were prepared as in panels b to e and subjected to click labeling. Particle yield was assessed by RT activity assay (79), and samples were titrated on TZM-bl indicator cells seeded in 15-well ibidi μ-slide angiogenesis dishes. 50 μM T-20 was added at 6 h p.i. to prevent second-round infection in the case of HIV-1*. Cells were fixed, permeabilized, and immunostained using a polyclonal rabbit antiserum raised against recombinant HIV-1 MA at 48 h p.i. Samples were imaged by SDCM. The percentage of infected cells was determined using Fiji software. The graphs show mean values and SD from six independent infection experiments using five independent particle preparations (*n* = 5,700 to 7,700 cells were counted per condition). Lines represent linear regression based on the mean values. (g) Relative infectivity of a virus preparation (% infected cells/mU RT) was determined as in panel f, and the values obtained for HIV-1*CA14^SiR^ were normalized to the value obtained for HIV-1* virus in the same experiment. All cells counted in panel f were used for quantification. The graph represents the mean value and SD from six independent experiments.

In order to determine fluorescence staining specificity, particle lysates of HIV-1* and HIV-1*CA14^CpK^ stained with Tet-SiR were analyzed by SDS-PAGE followed by in-gel fluorescence measurement (Fig. 3a). The resulting image revealed a distinct SiR labeled band corresponding to a mass of approximately 24 kDa and weak bands corresponding in size to CA-containing precursors in the case of HIV-1*CA14^SiR^, whereas fluorescent protein bands were undetectable for the HIV-1* control (Fig. 3a, bottom). Subsequent immunoblotting of the scanned gel using antiserum raised against CA ensured that similar amounts of virus particles had been loaded for wt and the CA14 variant (Fig. 3a, top). These results indicated specific GCE-dependent labeling of CA *via* amber suppression at position 14 of HIV-1 CA.

To test efficiency of SiR staining, labeled particles adhered to a glass chamber slide were fixed, permeabilized, and immunostained with antiserum raised against HIV-1 CA to validate that detected signals corresponded to virus particles and imaged by confocal microscopy (Fig. 3b and c). Confocal micrographs were recorded in the channels corresponding to the CA (IF) stain (green) and direct CA labeling with SiR (magenta). Regions of interest (ROIs) corresponding to the position of virus particles were defined based on CA(IF) signals. Measurement of SiR fluorescence intensities in these ROIs revealed only weak background staining in the case of HIV-1* (Fig. 3b). In contrast, distinct SiR signals colocalizing with CA(IF) punctae were detected for HIV-1*CA14^SiR^ (Fig. 3c). Quantitative analyses of images from multiple independent experiments confirmed this visual impression (Fig. 3d and e). Only ~8.5% of HIV-1* particles were classified as SiR positive, with fluorescence intensities only slightly above the background level (~1,000 AU; Fig. 3d). In contrast, >95% HIV-1*CA14^SiR^ particles displayed clear SiR staining, with a mean fluorescence intensity of ~15,000 AU (Fig. 3e). Variation in SiR fluorescence intensities between individual particles is expected, since particle size and CA content of HIV-1 virions vary, with ~1,700 to 3,100 CA monomers estimated per particle (41). Beyond that, the range of SiR signal intensities observed also indicated a range of click labeling efficiencies. Despite variable staining intensities within the preparation, the vast majority of HIV-1*CA14^CpK^ particles could be efficiently click labeled with SiR, attaining fluorescence intensities suitable for fluorescence microscopy of infected cells.

To test the effect of introducing a synthetic fluorophore at position 14 on CA functionality, the infectivity of click-labeled particles was assessed by titration of labeled particles on TZM-bl cells, followed by immunostaining against the HIV-1 matrix protein (MA) to identify infected cells. As shown in Fig. 3f and g, relative infectivity of HIV-1*CA14^SiR^ was only mildly reduced by an average of ~2-fold compared to HIV-1*. This moderate reduction in infectivity represented a substantial improvement compared to previous genetically labeled derivatives in the absence of complementation (18, 27–29). Thus, minimal invasive labeling by GCE allows direct labeling of HIV-1 CA without requiring complementation with wt virus.

### Detection of click-labeled HIV-1 in infected cells.

**FIG 4:**
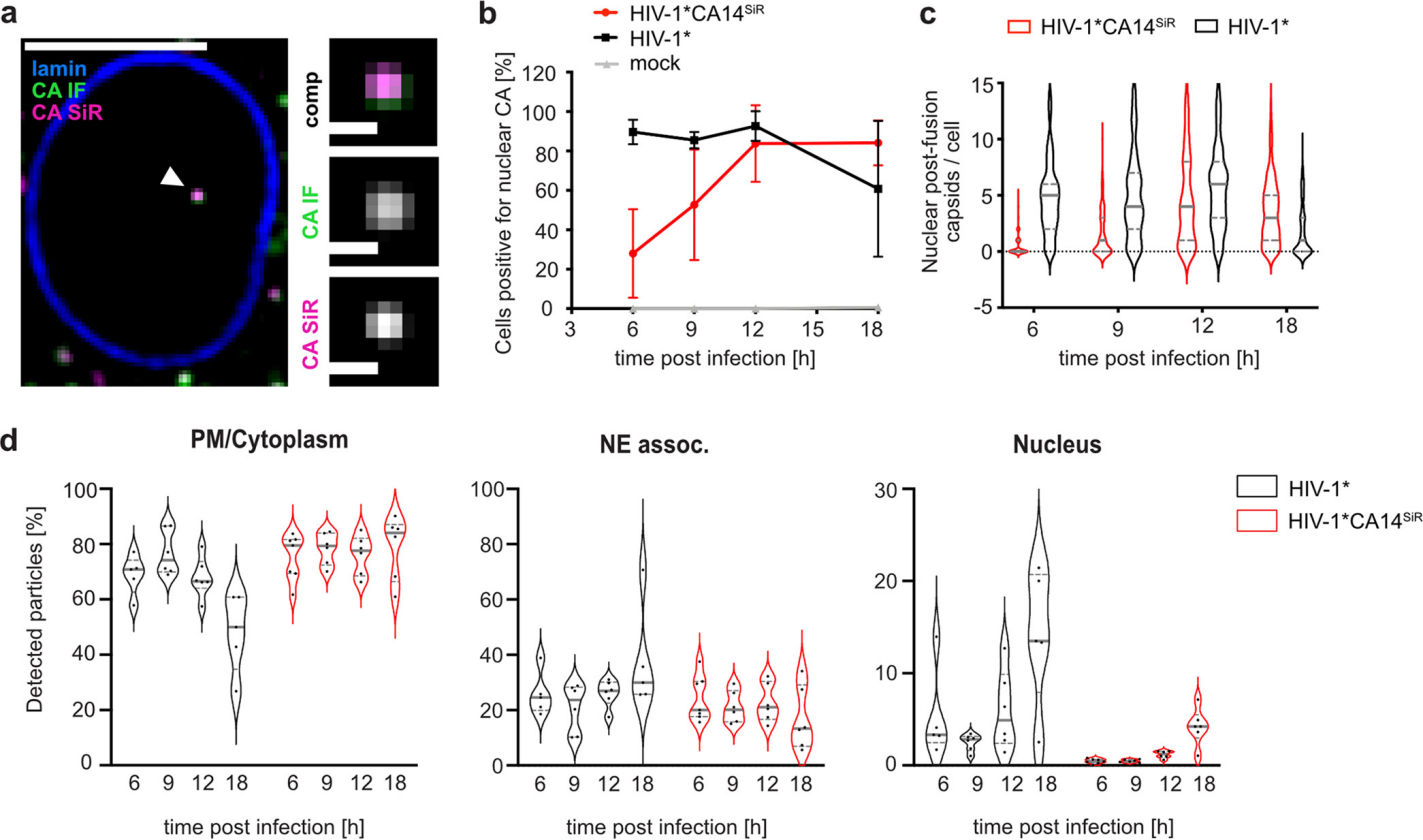
Detection of CA in the nucleus of infected HeLa-derived cells. TZM-bl cells were infected with HIV-1* or HIV-1* CA14^SiR^ particles (~MOI, 0.8), treated with 15 μM PF74 for 1 h before fixation, fixed at 6, 9, 12, and 18 h p.i. and imaged by SDCM. (a) Single z slice of a representative cell infected with HIV-1* CA14^SiR^ at 18 h p.i. and one enlarged z slice are shown. Scale bars: 10 μm (cell) and 1 μm (enlargement). Mean filter and background subtraction was applied for clarity. The image shows a representative image from one of three independent experiments. See Fig. S5 for additional data. (b to d) Infection time course of click-labeled HIV-1* CA14^SiR^ compared to HIV-1*. Quantification of cells containing nuclear CA-positive objects at the indicated times postinfection for HIV-1* [black; CA(IF)], HIV-1* CA14^SiR^ [red; CA(IF)/CA(SiR)] and noninfected control ]gray; CA(IF)]. Mean values and SD from three independent experiments are shown (*n* > 115 cells per time point). (c) Number of nuclear CA foci per cell determined for cells infected with HIV-1* (black) or HIV-1* CA14^SiR^ (red) at the indicated time points; *n* > 120 cells were analyzed per sample. The median and interquartile lines are indicated in gray. (d) Localization of particles within the cell for HIV-1* (black) and HIV-1* CA14^SiR^ (red). The proportion of total particles per cell detected at the PM or in the cytoplasm (PM/cytoplasm) at the nuclear envelope (NE assoc.) or inside the nucleus was determined at the indicated time points. Data from two of three independent experiments are shown. Graphs show median of analyzed field of views in red (*n* > 20 cells per condition) and interquartile lines.

Having established a suitable labeling strategy, we used labeled particles to infect and image target cells. Initial experiments were performed in the model cell line HeLa TZM-bl. Cells infected with HIV-1*CA14^SiR^ at a multiplicity of infection (MOI) of ~0.8 were fixed at 18 h postinfection (h p.i.). Immunostaining with antiserum against CA was performed under conditions that allow for immunodetection of cytosolic and nuclear complexes (17) to test whether detected SiR signals corresponded to HIV-1 particles. Labeled particles could be visualized by spinning disc confocal microscopy (SDCM) in the cellular environment (Fig. 4a; Fig. S4 and S5). Confocal images revealed punctate SiR signals in the cytosol, close to the nuclear envelope and within the nucleus of infected cells. Colocalization with CA(IF) staining confirmed that these signals represented entering viral structures (Fig. 4a; Fig. S4 and S5).

10.1128/mbio.01959-22.4 FIG S4 Detection of click-labeled HIV-1*CA14^SiR^ particles in TZM-bl cells. TZM-bl cells were infected with HIV-1* or HIV-1*CA14^SiR^, treated with 15 μM PF74 for 1 h before fixation at 8 h p.i., and immunostained against lamin A/C (blue) and HIV-1 CA (green) and SDCM imaged. (a) Representative maximum projections from cells infected with HIV-1* or HIV-1* CA14^SiR^. Enlargements of the boxed areas are shown below. Scale bars: 10 μm (cell) and 5 μm (enlargement). (b) Mean CA(SiR) intensities plotted against mean CA(IF) intensities of individual intracellular punctae for HIV-1* and HIV-1*CA14^SiR^. The graphs represent data from one of three independent experiments; *n* = 5 cells for HIV-1*CA14^SiR^ and *n* = 6 for HIV-1*. The threshold in the SiR channel was set to t = 1,000 AU indicated by the red line. Download FIG S4, TIF file, 0.8 MB. Copyright © 2022 Schifferdecker et al. 2022 Schifferdecker et al. https://creativecommons.org/licenses/by/4.0/ This content is distributed under the terms of the Creative Commons Attribution 4.0 International license.

10.1128/mbio.01959-22.5 FIG S5 Confocal micrographs of TZM-bl cells infected with HIV-1*CA14SiR particles. Infection was performed using an MOI~0.8 for 18 h p.i. Cells were fixed, immunostained against lamin A/C (blue) and HIV-1 CA (green) and imaged by SDCM. Images show a single z-slice through the middle of the cells. Arrowheads point towards nuclear CA(IF)/CA(SiR)-positive objects (i to vi). Enlargements of the indicated nuclear objects are shown to the right of the overview. Mean filter and background subtraction were applied to all images for clarity. Scale bars = 10 μm (overview) and 1 μm (enlargements). Download FIG S5, TIF file, 1.2 MB. Copyright © 2022 Schifferdecker et al. 2022 Schifferdecker et al. https://creativecommons.org/licenses/by/4.0/ This content is distributed under the terms of the Creative Commons Attribution 4.0 International license.

Next, TZM-bl cells infected with HIV-1* or HIV-1*CA14^SiR^ were fixed and analyzed for the presence of click-labeled subviral particles inside the nucleus at different time points after infection. Consistent with earlier results (17, 18, 27), we observed nuclear CA(IF)-positive foci in HIV-1*-infected cells as early as 6 h p.i. (Fig. 4b, black), while such signals were absent in noninfected cells (Fig. 4b, gray). Importantly, we detected SiR-positive complexes in the nucleus of HIV-1*CA14^SiR^-infected cells, with the vast majority also positive for CA(IF) (Fig. 4b, red). Nuclear entry appeared to be delayed for HIV-1*CA14^SiR^ compared to HIV-1* by 6 to 12 h. Nevertheless, samples infected with HIV-1*CA14^SiR^ displayed a comparable proportion of cells with detectable capsid-like objects in the nucleus as the HIV-1*-infected control at 12 h p.i. (Fig. 4b). The same was true for the number of nuclear objects detected per cell (Fig. 4c); at 12 h p.i., HIV-1*CA14^SiR^ reached an average of 4.58 ± 4.12 nuclear particles per cell, similar to HIV-1* with 5.91 ± 4.11.

Delayed detection of subviral complexes in the nucleus may be due to slower uptake, slower trafficking toward the nuclear envelope, delayed passage through the NPC, or a combination thereof. In order to distinguish between these possibilities, we extended the time-resolved quantification to objects in close vicinity to the nuclear envelope (Fig. 4d). This analysis revealed that the HIV-1*CA14^SiR^-derived subviral structures reached the nuclear envelope with similar kinetics as HIV-1* particles (Fig. 4d, NE assoc.). A comparable average proportion of CA-containing objects was detected at the nuclear envelope in both cases at 6 h p.i., while the numbers of nuclear capsids were lower for HIV-1*CA14^SiR^ at that time (Fig. 4d, Nucleus). In contrast, the highest proportion of HIV-1*CA14^SiR^ nuclear objects with 4.20 ± 1.80% was detected at 18 h p.i., while HIV-1* reached similar levels at 6 h p.i. This implies that the mechanistic action of the capsid in nuclear import underlies tight margins with respect to its biophysical properties.

### Characterization of nuclear CA^SiR^-containing complexes.

A long-standing question in the field of HIV-1 early replication is when and where genome uncoating takes place. The possibility to directly detect CA molecules clicked to a synthetic fluorophore enabled us to assess the amounts of CA associated with subviral complexes at different intracellular sites, without the influence of differential epitope accessibility or of a tag domain that potentially confers different properties to a subpopulation of CA molecules. However, comparing labeling intensities for nuclear, cytoplasmic, and extracellular particle-associated structures may be additionally confounded in diffraction-limited microscopy by the failure to resolve closely adjacent individual capsids. Clusters of nuclear capsids had indeed been observed by CLEM analyses in our previous study (17).

**FIG 5:**
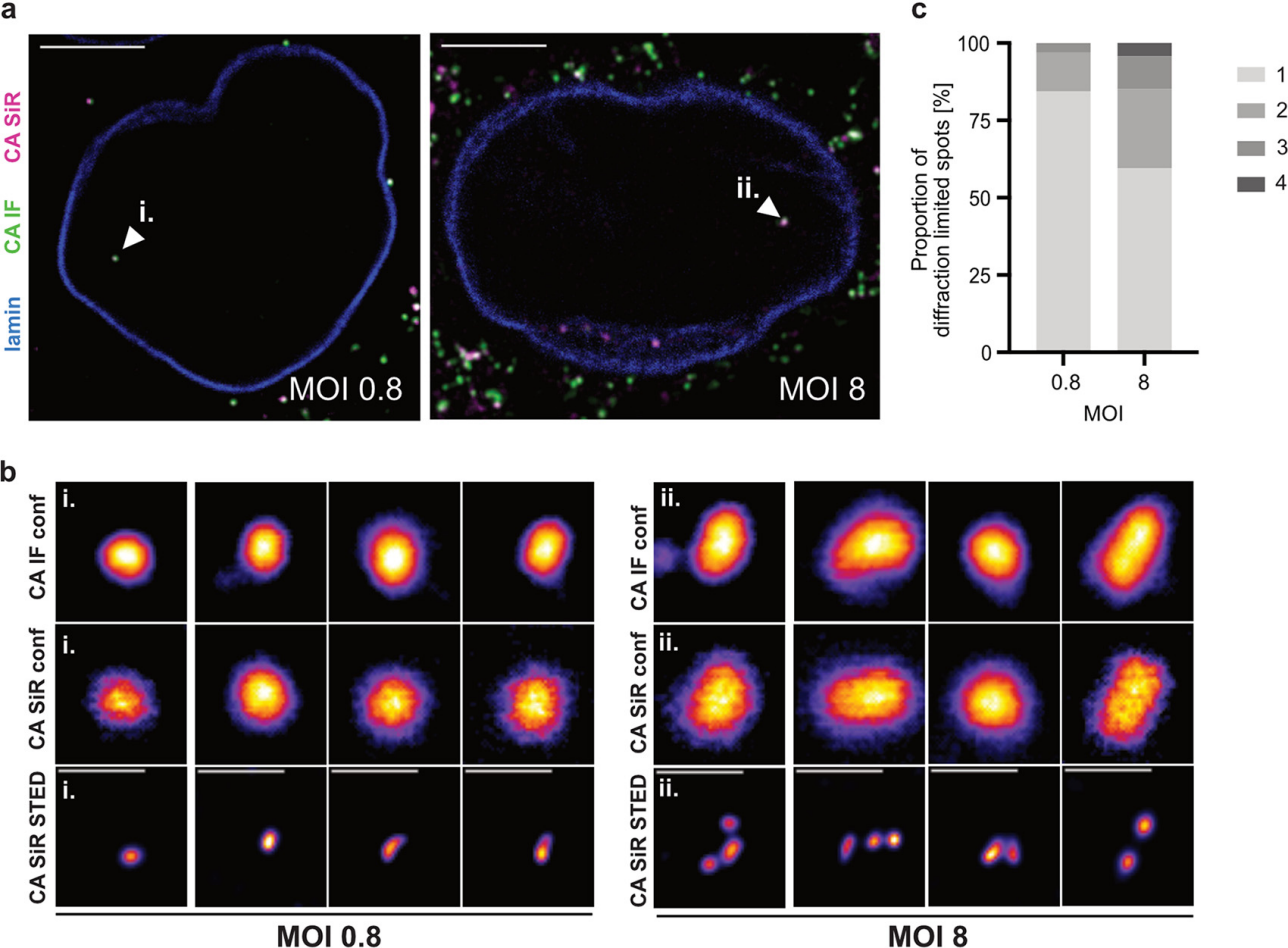
Dose dependent clustering of nuclear capsids in HeLa-derived cells. TZM-bl cells were infected with HIV-1* CA14^SiR^ at the indicated MOI, treated with 15 μM PF74 for 1 h before fixation at 18 h p.i., immunostained against CA (green) and lamin A/C (blue) and imaged using an Abberior STED microscope setup. Mean filter and background subtraction was applied to all images for clarity. (a) Micrographs of TZM-bl cells infected with an MOI of ~0.8 (left) or MOI of ~8 (right). Arrowheads indicate nuclear CA(IF)/CA(SiR)-positive objects shown enlarged in panel b. Scale bars = 10 μm. (b) Representative images of nuclear CA containing objects from cells infected with a low MOI (~0.8, left) or a high MOI (~8, right). CA(IF) and CA(SiR) were imaged in confocal mode (top and middle row, respectively). CA(SiR) images were also recorded in STED mode (bottom row). The figure shows four representative foci each from one of two individual experiments. Mean filter and background subtraction were applied. Scale bars = 500 nm. (c) Diffraction-limited nuclear foci were analyzed by STED nanoscopy in cells infected with an MOI of 0.8 (*n* = 32 foci) or MOI of ~8 (*n* = 47 foci) and classified by the number of individual capsids per focus.

To determine whether nuclear cluster formation occurred under our conditions, we exploited the fact that the chemical dye conjugated to the capsid surface renders the modified virus suitable for superresolution microscopy. With a lateral resolution of <50 nm, STED nanoscopy allows visual separation of closely adjacent CA objects. TZM-bl cells were infected with HIV-1*CA14^SiR^ at two different MOIs. An MOI of ~0.8 corresponded to the conditions generally used in our experiments; a 10-fold higher virus dose (MOI, ~8) was applied in a parallel experiment to test for enhancement of capsid clustering. At 18 h p.i., cells were fixed, immunostained against CA, and imaged using a STED system in confocal and STED mode (Fig. 5). Nuclear CA(IF)/(SiR) double-positive objects were detected under both conditions (Fig. 5a, arrowheads). While these objects appeared as individual punctae in diffraction-limited micrographs from the IF and SiR channels at both MOIs (Fig. 5b, top and middle row), imaging of the SiR channel in STED mode revealed differences between individual punctae. Some diffraction-limited punctae in the nucleus represented individual capsid-like objects when imaged by STED (Fig. 5b, bottom left). In contrast, other punctae were resolved into small clusters of 2 to 4 closely apposed CA-containing objects by superresolution microscopy (Fig. 5b, bottom right), consistent with observations made by electron tomography (16, 17). A quantitative analysis of cluster sizes (Fig. 5c) revealed that the propensity for capsid clustering in the nucleus correlated with the amount of virus used for infection. Consistent with a normal distribution of infection events per cell at a given MOI, ~50% of those cells positive for nuclear punctae displayed only a single STED-resolved object at an MOI of 0.8, whereas all cells analyzed at an MOI of 8 comprised more than one STED resolved nuclear object, with up to 15 objects identified in one nucleus (Fig. S6). Accordingly, at an MOI of ~0.8, the vast majority of punctae (~88%) corresponded to individual capsid-like objects in the nucleus, and clusters of more than two objects were not observed. In contrast, the majority of cells analyzed comprising more than one nuclear object displayed clustered particles, and almost half of the nuclear punctae (~43%) corresponded to clusters of 2 to 4 objects when cells were infected with an MOI of ~8. We conclude that nuclear capsid clustering is rarely observed at the MOI of 0.8 used throughout this study and that nuclear clustering appears not to be a random event. The previously observed capsid clustering in distinct nuclear positions as well as larger clusters occurred preferentially at high MOI, where a higher proportion of cells contained multiple nuclear particles.

10.1128/mbio.01959-22.6 FIG S6 Number of individual STED resolved particles per nucleus. TZM-bl cells were infected with HIV-1*CA14^SiR^ at the indicated MOI, treated with 15 μM PF74 for 1h before fixation at 18 h p.i. and immunostained against CA and lamin A/C as described in Materials and Methods. CA(IF) was imaged in confocal mode, CA(SiR) in confocal and STED mode. Cells displaying at least one diffraction limited CA signal within the nucleus were analyzed in STED mode to determine the total number of individual particles. Dots represent individual cells (MOI, 0.8: mean = 2.4, *n* = 16; MOI of 8: mean = 6.8, *n* = 11). Black: exclusively individual particles detected; red: cell comprises at least one cluster. Graphs show median and interquartile lines in gray. Download FIG S6, TIF file, 0.1 MB. Copyright © 2022 Schifferdecker et al. 2022 Schifferdecker et al. https://creativecommons.org/licenses/by/4.0/ This content is distributed under the terms of the Creative Commons Attribution 4.0 International license.

We next proceeded to SiR fluorescence intensity measurements, comparing the signal intensity of extranuclear HIV-1 particles to that of subviral structures in the nucleus. Staining of the plasma membrane with mCLING ATTO488 before infection revealed that under our conditions most cell-associated particles in the cytosolic region represented virions present in endosomes, corresponding to a prefusion state of the virus (Fig. S7a to c). To ensure that these extranuclear punctae represented single objects, cytoplasmic foci were analyzed in STED mode. We found that ~95% (*n* = 79) of analyzed punctae corresponded to an individual object, while only ~5% (*n* = 4) of these foci were resolved into two adjacent objects by nanoscopy (Fig. S8).

10.1128/mbio.01959-22.7 FIG S7 The majority of HIV-1*CA14SiR particles in the cytosol of HeLa-derived cells is detected in endosomal vesicles. (a) To test viability of cells in in the presence of mCLING ATTO488, the compound was titrated on TZM-bl cells. Cells were incubated for 30 min at 16°C and before counting using an automated cell counter (Greiner Bio-one). Data represent the mean of one experiment performed in duplicate. (b and c) Following incubation with 2 μM mCLING ATTO488 at 16°C for 30 min, TZM-bl cells were infected with 10 μU RT/cell at 37°C, fixed at 3 h p.i. with 4% PFA + 0.2% GA and imaged by SDCM. (b) Representative image from one experiment (single z-slice through the middle of the cell). Enlargement of the boxed area of the cell (right) shows colocalization of mCLING ATTO488 (green) and CA SiR (magenta) signals. Scale bars = 10 μm (overview) and 5 μm (enlargement). (c) Quantitation of mCLING ATTO488-positive (98.78%) and-negative (1.22%) CA SiR objects (total *n* = 2,606, 5 cells) as described in Materials and Methods. (d) Quantitation of CA SiR signals of mCLING ATTO488-positive (*n* = 62, mean = 41,397 ± 10,909 AU) and-negative (*n* = 17, mean = 27,655 ± 5,812 AU) objects in the cytosol. Graphs represent mean values and SD. Download FIG S7, TIF file, 0.6 MB. Copyright © 2022 Schifferdecker et al. 2022 Schifferdecker et al. https://creativecommons.org/licenses/by/4.0/ This content is distributed under the terms of the Creative Commons Attribution 4.0 International license.

10.1128/mbio.01959-22.8 FIG S8 STED nanoscopy of CA(IF)/CA(SiR) double-positive objects in the cytoplasm of TZM-bl cells. (a) TZM-bl cells were infected with HIV-1*CA14^SiR^ (MOI ~0.8) for 18 h, fixed and immunostained against HIV-1 CA, and imaged using an Abberior STED system. Cytoplasmic objects were detected by CA(IF) (top) and CA(SiR) (middle). Diffraction limited double-positive foci localized in confocal mode were imaged in STED mode in the SiR channel (bottom). Mean filter and background subtraction was applied to all images for clarity. Scale bars = 1 μm. (b) The number of individual capsids per diffraction limited spot (*n* = 83) was determined from STED images. Approximately 95% (*n* = 79) of analyzed objects corresponded to an individual particle, while ~5% (*n* = 4) of foci were resolved into two objects by nanoscopy. Download FIG S8, TIF file, 0.3 MB. Copyright © 2022 Schifferdecker et al. 2022 Schifferdecker et al. https://creativecommons.org/licenses/by/4.0/ This content is distributed under the terms of the Creative Commons Attribution 4.0 International license.

**FIG 6:**
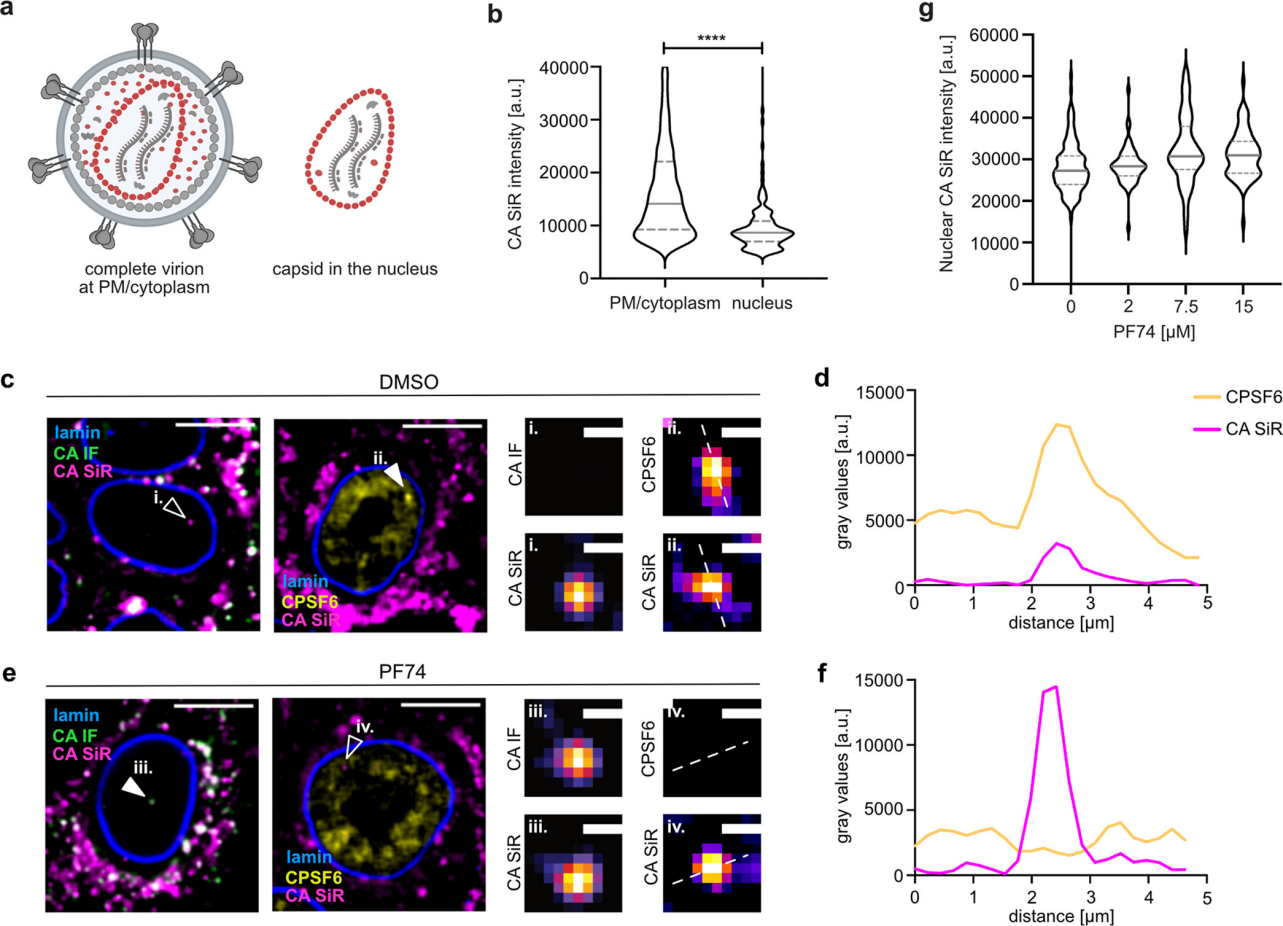
Largely intact capsids are detected in the nucleus of HeLa-derived cells. TZM-bl cells were infected with HIV-1* or HIV-1* CA14^SiR^ (MOI, 0.8), treated with 15 μM PF74 for 1 h before fixation at 18 h p.i., and imaged by SDCM. (a) Scheme of the relative CA content in complete virions (~2,400 CA) on glass/plasma membrane or in endosomes in the cytosol. Postfusion capsids contain only the CA molecules incorporated into the mature capsid lattice (~1,500 CA). Image created with BioRender. (b) Quantification of CA(SiR) intensities associated with CA(IF)-positive objects at the indicated localizations. Data from three independent experiments are shown. Cells from 7 fields of view were analyzed (n_particles_ = 6,441 PM/cytoplasm, 135 nucleus). Lines indicate median values (PM/cytoplasm: 17,649.22 ± 11,663.47; nucleus: 9,835.08 ± 5,708.14) and interquartile range. Significance was determined by two-tailed Student`s *t* test (****, *P* < 0.001). (c to g) TZM-bl cells were infected with HIV-1*CA14^SiR^, treated with DMSO (c and d) or 15 μM PF74 (e and f) for 1 h before fixation at 18 h p.i. Cells were immunostained against lamin A/C and HIV-1 CA or CPSF6 and subsequently imaged by SDCM. Scale bars = 10 μm (cells) and 1 μm (enlargements). (d) Quantification of intensity profile measured across the dashed line in panel cii for CA(SiR) and CPSF6. (f) Quantification of intensity profile measured across the dashed line in panel eiv for CA (SiR) and CPSF6. (g) Quantification of CA(SiR) intensities of nuclear objects. TZM-bl cells were treated with DMSO (*n* = 115) or 2 μM (*n* = 58), 7.5 μM (*n* = 110), 15 μM (*n* = 69) PF74 for 1 h prior fixation at 17 h p.i. Graphs show median values (DMSO: 2,7255; 2 μM: 2,8334.51; 7.5 μM: 3,0749.45; 15 μM: 3,0957) and interquartile range.

As illustrated by the cartoon in Fig. 6a, complete virions comprise on average ~2,400 CA molecules, while only ~1,200 to 1,500 of these are part of the mature fullerene capsid (5, 41) that represents a postfusion state. Assuming equal click-labeling efficiency of CA14^CpK^ for molecules that are part of the mature lattice and those that remain free in the virion volume, the average SiR intensity of complete capsids is expected to correspond to ~50 to 60% of the average intensity of complete virions from the same preparation. We infected TZM-bl cells at an MOI of 0.8 and quantified the SiR intensity of virions attached to the cell or in the cytosolic region (and thus mostly in endosomes) as well as nuclear punctae (Fig. 6b). As a control, we compared SiR intensities of the small proportion of mCLING-negative (postfusion) structures in the cytoplasmic region with that of mCLING-positive (endosomal) objects. This analysis revealed an average SiR intensity of ~66% for mCLING-negative compared to mCLING-positive objects (Fig. S7d), indicating that we can reliably differentiate between complete virions and postfusion complexes based on intensity of CA staining. The average SiR intensity of >6,000 cell-attached and (mostly) endosomal particles in the cytosolic region exhibited an average of 17,649 arbitary units (AU). In contrast, the SiR intensity of >100 nuclear subviral structures averaged 9,835 AU (Fig. 6b), i.e., ~56% of the average intensity of complete virions, similar to the predicted relative CA content of the mature capsid and similar to the intensity of mCLING-negative cytoplasmic objects. Based on these findings, we conclude that the CA(SiR)-containing objects in the nuclei of these cells correspond approximately to the full CA complement of the mature capsid.

The small molecule inhibitor PF74 (42) binds to the HIV-1 capsid in a pocket overlapping the binding sites for the FG motifs of various nucleoporins and for the nuclear host protein CPSF6 (43–45). This compound inhibits HIV-1 replication by multiple mechanisms, with effects on different replications steps reported based on PF74 concentration and time of addition (reviewed in reference 46). Treatment with high concentrations of PF74 has been reported to destabilize the capsid (18, 47), but data obtained in recent studies argued against a PF74-induced loss of CA from nuclear complexes (17) and even showed an increased CA(IF) signal (48). These findings are consistent with a stabilizing effect of the compound on the capsid lattice observed for isolated capsids after initial opening (49, 50) and for *in vitro* assembled CA-nucleocaspid particles (51). Since results obtained by immunodetection may be influenced by differential CA epitope exposure, we revisited this issue employing direct CA labeling. TZM-bl cells were infected with HIV-1*CA14^SiR^ particles for 17 h and then treated with 2 to 15 μM PF74 or DMSO for 1 h, followed by fixation, permeabilization, methanol extraction, and SDCM imaging. CA(SiR) punctae detected in the nucleus of DMSO control cells were positive for CPSF6 (Fig. 6cii). Intensity profile measurement revealed clear colocalization of both signals indicating that direct capsid labeling did not affect association with CPSF6 (Fig. 6d). In accordance with earlier results (17, 48), CA(SiR)-positive subviral complexes in nuclei of cells subjected to PF74 treatment lacked CPSF6 association (Fig. 6e and f). Their mean CA(SiR) intensity remained unaltered, however, indicating that the capsid remains largely stable in the presence of PF74 concentrations ranging from 2 to 15 μM (Fig. 6g).

### Detection of directly labeled HIV-1 capsids in primary cells.

**FIG 7:**
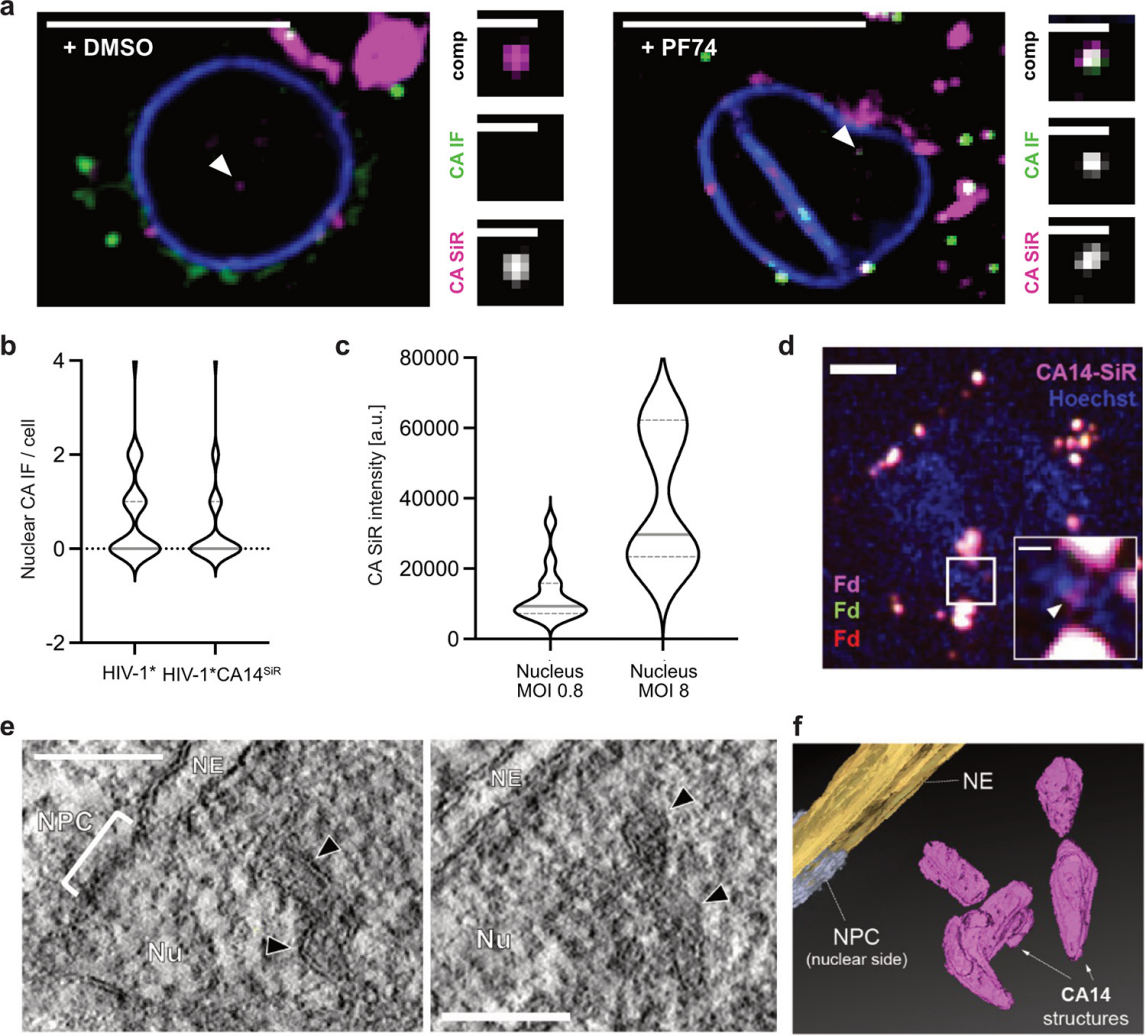
Largely complete click-labeled capsid structures detected in the nucleus of primary CD4^+^ T cells and T cell line. (a) Activated CD4^+^ T cells were infected with HIV-1*CA14^SiR^ (MOI, ~0.8), treated with DMSO/PF74 treatment for 1 h before fixation at 24 h p.i., and extracted with methanol. Samples were immunostained against CA (green) and lamin A/C (blue). Images show a single z slice through the cell. Enlargements show the particle marked by the arrowhead. Scale bars: 10 μm (overview) and 1 μm (enlargement). (b) Data analyzed from the experiment outlined in (a). The graph shows the number of CA-positive foci per nucleus in cells infected with HIV-1* (*n* = 35 cells, mean = 0.85) or HIV-1*CA14^SiR^ (*n* = 73 cells, mean = 0.51). Pooled data from 6 different blood donors are shown. Gray lines show median and interquartile lines. (c) CA(SiR) intensities of nuclear objects in infected and activated CD4^+^ T cells at an MOI~0.8 (*n* = 13; mean = 12,485 ± 7,445 AU) and an MOI of ~8 (*n* = 7; mean = 39,502 ± 18,025 AU). MOI was determined in TZM-bl cells. Gray lines show median and interquartile lines. (d to f) Nuclear cone-shaped capsids detected by CLEM-ET. SupT1 cells were treated with 1 μM aphidicolin (APC) for 16 h to prevent cell division, before infection with HIV-1*CA14^SiR^ virions (2.3 μU RT/cell, corresponds to an MOI of ~0.4 determined in TZM-bl cells). At 24 h p.i., cells were cryoimmobilized by high-pressure freezing, freeze substituted, and further processed for CLEM and ET as described in Materials and Methods. (d) SDCM image of a 250-nm-thick resin section of the cell infected with HIV-1*CA14^SiR^ virions (magenta), poststained with Hoechst (blue) and decorated with multi-fluorescent fiducials (Fd) for correlation. The arrowhead in the enlargement of the boxed region indicates a CA(SiR) signal within the Hoechst-stained nuclear region. Scale bars = 1 μm (overview) and 200 nm (enlargement). (e) Computational slices through tomographic reconstructions at the correlated region boxed in panel d with views highlighting the presence of clustered capsid-reminiscent structures (black arrowheads) in the nuclear region. Nu, nucleus; NPC, nuclear pore complex; NE, nuclear envelope. Scale bar = 100 nm. (f) Segmented and isosurface rendered structure of the cones detected in panel e. Magenta: capsid; yellow: NE; cyan: NPC (cryo-EM map of NPC: EMD-11967 [16]). See also Movie S1.

To validate our results in a physiologically relevant cell type, primary human CD4^+^ T cells from healthy blood donors were infected, subjected to IF staining against CA, and imaged by SDCM at 24 h p.i. (Fig. 7a and Fig. S9). We readily detected nuclear subviral SiR-positive structures in HIV-1*CA14^SiR^-infected cells, indicating that nuclear replication complexes retained CA also in these primary cells (Fig. 7a). Consistent with prior observations made in T cell lines (22), the majority of SiR-positive objects were not associated with CA(IF) signals (9/11 particles; Fig. 7a, *left* and Fig. S9a) when fixation and immunostaining were performed under standard conditions. As outlined above, treatment with 15 μM PF74 for 1 h dissociates the large clusters of CPSF6 from nuclear subviral complexes. We observed that this in turn renders nuclear CA accessible for IF detection in T cells, presumably by exposure of CA epitopes upon CPSF6 displacement (17). Accordingly, brief PF74 treatment allowed for detection of CA(IF) signals colocalizing with nuclear CA(SiR) punctae (13/16; Fig. 7a, *right* and Fig. S9b). We conclude that the direct CA-labeling strategy presented here overcomes technical artifacts that hamper IF analyses.

10.1128/mbio.01959-22.9 FIG S9 Confocal micrographs of primary CD4+ T cells infected with HIV-1*CA14SiR. (a and b) Cells were infected (MOI, 0.8, determined in TZM-bl cells) and subsequently treated with DMSO (a) or 15 μM PF74 (b) for 1 h before fixation at 24 h p.i., immunostaining against CA (green) and lamin B1 (blue) and SDCM. Images show a single z-slice through the middle of the cell; two representative examples are shown per condition. Arrowheads point towards nuclear CA(IF)/CA(SiR) double-positive objects. Enlargements of the indicated complexes are shown on the right. Mean filter and background subtraction was applied on each image for clarity. Scale bars = 10 μm (overview) and 1 μm (enlargements). Download FIG S9, TIF file, 0.9 MB. Copyright © 2022 Schifferdecker et al. 2022 Schifferdecker et al. https://creativecommons.org/licenses/by/4.0/ This content is distributed under the terms of the Creative Commons Attribution 4.0 International license.

Further quantitative analyses using primary CD4^+^ T cells prepared from six blood donors revealed similar numbers of nuclear capsid structures in cells infected with HIV-1*CA14^SiR^ compared to cells infected with HIV-1* at 24 h p.i. (Fig. 7b). SiR intensity measurements were only performed for intranuclear objects in this case, since high background due to SiR accumulation in the narrow cytoplasm of T cells precluded reliable analysis of individual particles in the extranuclear region. Quantitation of SiR intensities of nuclear punctae in cells infected with an MOI of ~0.8 yielded similar average intensities as measured in TZM-bl cells (mean = 12,485 AU), indicating the presence of a complete or nearly complete mature capsid in the nuclear complexes in primary T cells as well (Fig. 7c). Cells infected with an MOI of ~8 displayed higher CA(SiR) intensities of diffraction-limited nuclear objects (mean = 39,502 AU), suggesting intranuclear clustering of capsids, as observed in TZM-bl cells (Fig. 5).

Our findings from CA(SiR) intensity measurements argued for the presence of a full capsid complement at subviral structures in the nucleus. These data strengthen conclusions from several recent studies suggesting that the mature capsid lattice may be completely or largely intact on nuclear subviral objects (16–18). However, fluorescence signals do not yield information on the architecture of nuclear CA14^SiR^-containing objects. Therefore, we complemented our analyses by performing CLEM of infected SupT1 T cells. In order to maximize the number of nuclear objects, infection was synchronized by the attachment of particles to the cells for 3 h at a low temperature (16°C) to prevent particle uptake by membrane fusion or endocytosis (52, 53). Virus entry was initiated by temperature shift to 37°C. At 24 h post transfection (h p.t.), specimens were prepared by high-pressure freezing and freeze substitution. Resin sections (250 nm thick) were subjected to SDCM in order to localize CA(SiR)-containing structures, followed by correlative electron tomography (CLEM-ET) analysis. CA(SiR)-positive objects could be identified by SDCM in the sections (Fig. 7d), demonstrating that the brightness of signals derived from direct CA(SiR) labeling is sufficient for CLEM detection of cytosolic and nuclear (sub)viral structures. ROIs were defined based on the SiR signals and subjected to correlative ET analysis. Fig. 7e shows an exemplary tomogram obtained from a ROI located within the nucleus. It reveals several closely apposed electron-dense structures at the position of the SiR label, whose shape and dimension match those of intact or largely intact mature HIV-1 capsids (Fig. 7f and Movie S1). Such structures were recently identified in nuclei of infected cells by CLEM using fluorescently labeled HIV-1 IN as an indirect marker for subviral structures (16, 17) and were interpreted as capsid shells solely based on their morphology. Here, we demonstrated that these capsid-resembling structures colocalize with nuclear foci comprising a high number of click-labeled CA molecules. We thereby provide direct evidence that the cone-shaped objects are complete or largely complete HIV-1 capsids that have entered the nucleus presumably through the NPC of infected and cell cycle-arrested cells.

10.1128/mbio.01959-22.10 MOVIE S1 CLEM-ET analysis of nuclear HIV-1*CA14SiR capsid-like structures in T cells. Related to Fig. 7e and f. Shown is a segmented and isosurface rendered tomographic reconstruction to highlight the morphology of several clustered capsid-related structures visualized inside the nucleus of an infected SupT1 T cell (upon APC treatment), correlated with the position of a CA(SiR) signal. Download Movie S1, AVI file, 18.5 MB. Copyright © 2022 Schifferdecker et al. 2022 Schifferdecker et al. https://creativecommons.org/licenses/by/4.0/ This content is distributed under the terms of the Creative Commons Attribution 4.0 International license.

## DISCUSSION

Here, we present a direct labeling approach for HIV-1 CA that yields infectious and morphologically mature viral particles. The minimally invasive GCE/click labeling approach used here represents an ideal strategy for the versatile labeling of genetically fragile viral capsid proteins in principle, but its potential for virus imaging has not been exploited so far. The combination of GCE and subsequent functionalization of a viral capsid protein by click chemistry has previously only been applied to the nonenveloped adeno-associated virus (AAV) (e.g., references 54, 55). However, the capsid of AAV, unlike HIV-1 CA, can also tolerate peptide insertions and larger modifications (56–59). Here, we demonstrate that GCE in conjunction with click labeling can also be applied to an enveloped virus with a highly multifunctional and genetically fragile capsid protein that needs to form a closed fullerene lattice to be infectious. We found that HIV-1 CA tolerates chemical modification of the exposed amino acid residue A14. This position is located close to the central, dynamic pore in the CA hexamer lined by positively charged R18 and K25 rings (60). Replacement of A14 and E45 by cysteine residues for disulfide cross-linking CA subunits was previously shown to allow CA hexamer assembly and was used to study the capsid hexamer structure (61). This mutant retained binding to host cell proteins Nup153 and CPSF6, as well as to dNTPs and PF74 (60, 62), consistent with functional replacement of A14 by a nonnatural CpK residue in the current study.

All previously described genetic tagging strategies for HIV-1 CA (18, 27–29) required complementation with a molar excess of wt protein or virus. Since the mature HIV-1 capsid is assembled from approximately half of the ~2,500 CA molecules packaged in the virion (30, 31), it cannot be ascertained in this case whether the subset of genetically tagged CA molecules is an integral part of the mature capsid lattice. Conceivably, tagged or modified CA molecules may be excluded from the mature capsid shell or may be irregularly inserted. Signal intensity changes of CA fusion proteins in early HIV-1 replication may thus also depend on the relative incorporation of these proteins into the mature capsid lattice. In contrast, we found that the strategy described here allowed genetic labeling of HIV-1 CA in the proviral context and retaining almost full infectivity in the absence of wild-type complementation.

The detection of a label covalently attached to CA is independent of cellular context, sample treatment, or exposure of CA epitopes. Thereby, the method overcomes limitations of IF detection that had previously resulted in different conclusions regarding the presence of CA on subviral complexes. The use of synthetic dye molecules also renders the labeling strategy compatible with a wide range of fluorescence imaging approaches, including live-cell microscopy, correlative imaging, and superresolution fluorescence microscopy techniques (63).

Our approach allowed for direct, quantitative analysis of CA-containing objects and CA amounts associated with viral complexes in microscopic images of infected cells. While time-lapse experiments showed some delay in nuclear import kinetics for labeled capsid-like objects, the infectivity of highly labeled preparations was reduced by only 2-fold, and the number of nuclear objects reached was similar to that detected in cells infected with wt virus. Thus, site-specific introduction of a synthetic fluorophore can be compatible with capsid functionality in HIV-1 postentry processes. The delay in nuclear accumulation appears to be mainly caused by slower trafficking through the NPC, possibly due to the additional mass of the ncAA and label, given that the unmodified HIV-1 capsid already approaches the size limit even of dilated NPCs (16). Another, not mutually exclusive, possibility is that ncAA incorporation and/or the attached SiR dye affects pliability of the capsid structure that might be required for efficient and fast passage through the NPC.

CA amounts approximately corresponding to the full complement of a mature capsid were found to be associated with cytoplasmic postfusion objects and with subviral complexes in nuclei of a HeLa-derived cell line and primary human CD4^+^ T cells, also upon inhibition of cell division by aphidicolin treatment. By applying correlative imaging, we provide direct evidence that nuclear complexes positive for directly labeled CA indeed represent HIV-1 capsids or capsid-like remnants. Taken together, these results argue against (partial) capsid uncoating prior to entering the nucleoplasm, as had been concluded earlier based on low or lacking CA IF signals associated with nuclear subviral complexes in certain cell types (e.g., references 15, 21–23), or based on the loss of the fluorescently labeled capsid binding protein CypA at the nuclear envelope (12–14). The apparent discrepancy between these previous IF results and data from direct CA quantification may be explained by differential accessibility of CA epitopes under different IF conditions. The indirect label CypA, on the other hand, might be displaced from capsids at the nuclear pore, possibly by competition between fluorescent CypA and the outer NPC protein Nup358, which also carries a binding site for the CypA binding loop of CA (64). Our data suggest nuclear capsid uncoating in a model cell line, as well as in primary T cells, in agreement with recent findings from us and others, which indicated that the nuclear pore channel is wider than previously assumed, allowing HIV-1 capsids to pass the intact NPC (16) and that HIV-1 uncoating occurs after nuclear import (18–20, 65, 66), apparently by separation of the viral genome from a broken capsid remnant (17).

Small clusters of CA-positive objects were detected by STED nanoscopy in nuclei of TZM-bl cells and T cells, consistent with the reported detection of nuclear clusters containing multiple HIV-1 replication complexes (48), multiple viral genomes (67), or even several intact or partly intact capsid-like structures (17) in various cell types. Our analyses revealed that the observed clustering is dependent on the amount of virus used for infection. Most nuclear signals represented single capsids at a lower MOI, whereas frequent clustering was observed at high MOI. This observation suggests that capsids enter the nucleus individually but traffic via a limited number of routes and accumulate at defined sites of uncoating. This raises the question whether HIV-1 capsids use a “specialized” subset of nuclear pores for nuclear entry; the answer would not only be relevant in the context of HIV-1 replication but also with respect to an understanding of the nuclear import process. Intracellular Nup levels and presumably NPC composition have been reported to influence HIV-1 replication (68), but compositional and structural variability of NPCs between different cell types, or within an individual cell, is incompletely understood (reviewed in reference 69). The route, mechanism, and functional consequences of intranuclear trafficking of HIV-1 complexes also warrant further analysis. Growing evidence from recent studies suggests that incoming viral replication complexes accumulate at nuclear speckles in a CA- and CPSF6-dependent manner and that reverse transcription may only be completed near the site of integration (14, 18, 66, 67). Combining the direct CA labeling described here with the recently developed fluorescence detection of the reverse transcribed genome (17, 70) will provide us with the possibility to study the uncoating process in more detail using a combination of confocal imaging, nanoscopy and correlative imaging.

The direct labeling approach also allowed us to investigate the effect of the capsid inhibitor PF74 (42), whose detailed mode of action is still under investigation. We found that displacement of CPSF6 from nuclear subviral structures was not accompanied by a loss of CA signal. This finding disagrees with the recently reported rapid CA dissociation from nuclear complexes upon PF74 addition that was based on imaging of HIV-1 particles containing enhanced GFP-CA (eGFP-CA) complemented by a molar excess of wt CA (18). The apparent discrepancy may suggest that the subset of eGFP-tagged CA molecules is not an integral part of the mature capsid lattice, resulting in premature loss of the labeled molecules. Our findings are in line with the observation of other recent studies where PF74 treatment did not lead to a loss of the CA IF signal on nuclear complexes but rather enhanced immunostaining efficiency (17, 48) and with *in vitro* findings that indicated breach of lattice integrity by PF74 but stabilization of the remaining lattice (50, 71).

In conclusion, direct click labeling of HIV-1 CA is a versatile approach that substantially expands the possibilities to study the early events in HIV-1 replication with high temporal and/or spatial resolution using advanced fluorescence microscopy methods. Application for HIV-1 CA demonstrated its usefulness for genetically fragile structural proteins and provided direct proof that the capsid stays largely intact upon passage of the subviral complex into the nucleus. Furthermore, it directly identified nuclear capsid-like structures that morphologically resembled the virion capsid by CLEM-ET. The variety of synthetic fluorophores that can be combined with GCE and click labeling enables rapid adaptation of the approach to microscopic single-molecule techniques such as STORM (72) and MINFLUX (73). It can also be combined with labeling of proteins of the viral replication complex and of the viral genome, which is a subject of ongoing studies. Beyond fluorescence imaging, CA modification by GCE offers possibilities for site-specific incorporation of other functionalized ncAAs, e.g., benzoyl-phenylalanine (74) for photo-cross-linking, together with pulldown experiments for the detection of capsid-host protein interactions. The fact that the combination of GCE and click chemistry could successfully be applied to a notoriously genetically fragile capsid protein of an enveloped virus opens the perspective that this strategy may also advance and expand fluorescence labeling of a broad range of other viruses.

## MATERIALS AND METHODS

### Plasmids.

Plasmids were cloned using standard molecular biology techniques and verified by commercial Sanger sequencing (Eurofins Genomics). PCR was performed using Q5 High-Fidelity DNA polymerase (New England Biolabs) or Phusion DNA polymerase (New England Biolabs) according to the manufacturer’s instructions using primers purchased from Eurofins Genomics. Plasmid amplification was carried out in Escherichia coli Stbl2 (Thermo Fisher Scientific) cells.

HIV-1 plasmids were based on the proviral plasmid pNLC4-3 (35) that expresses the authentic genomic RNA from HIV-1_NL4-3_ (75) under the control of the cytomegalovirus promoter. To avoid unwanted ncAA incorporation into the virion component Vpr, the amber stop codon of the *vpr* ORF of pNLC4-3 was mutated into an opal stop codon (TGA) via site-directed mutagenesis. See Table 1 for sequences of primers used. PCR1 (primers Vpr_TGA_ a and Vpr_TGA_ b) and PCR2 (primers Vpr_TGA_ c and Vpr_TGA_ d) were performed in parallel to generate two overlapping single stranded PCR products. Using a combination of both products of these reactions as new templates, PCR3 with primers Vpr_TGA_ a and Vpr_TGA_ d resulted in PCR fragments comprising the respective mutation. These fragments were cloned into pNLC4-3 using unique PflMI/NheI restriction sites, resulting in pNLC4-3*. Virus produced from this plasmid was termed HIV-1*.

**TABLE 1:** Primers

Primer	Sequence
Vpr_TGA_ a	ctggcatttgggtcagggagtc
Vpr_TGA_ b	cagggctctagttcaggatctactggctc
Vpr_TGA_ c	gagccagtagatcctgaactagagccctg
Vpr_TGA_ d	gttctcttaatttgctagc
CA A14_BssHII_ fwd 1	cttgctgaagcgcgca
CA A14_TAG_ rev 1	agttctaggtgatatctactgatgtaccatttg
CA A14_TAG_ fwd 2	caaatggtacatcagtagatatcacctagaact
CA A14_ApaI_ rev 2	gccctgcaatttttggctatgtg

To allow for site-specific GCE, the codon for amino acid A14 of CA was mutated into TAG via overlap PCR. PCR1 (primers CA14_BssHII_ fwd 1, CA14_TAG_ rev 1) and PCR2 (primers CA14_TAG_ fwd 2, CA14_ApaI_ rev 2) were performed in parallel to generate two overlapping single-stranded PCR products. PCR3 with primers CA14_BssHII_ fwd 1 and CA14_ApaI_ rev 2 result in the PCR fragment comprising the mutation, which was subcloned into pNLC4-3* using unique BssHII/ApaI restriction sites, resulting in pNLC4-3*CA14^TAG^ (HIV-1*CA14^TAG^).

Plasmid pNESPylRS-eRF1dn-tRNA (Schifferdecker et al., unpublished data) is based on pEA168 (76), kindly provided by Eyal Arbely, Ben-Gurion University of the Negev, Israel), a eukaryotic vector that comprises expression cassettes for two proteins and four tRNA molecules. The coding sequence for a modified pyrrolysine tRNA synthetase was cloned from plasmid tRNA^Pyl^/NESPylRS^AF^ (37) into the CMV promoter driven expression cassette of pEA168 using HindIII/XbaI restriction sites, resulting in plasmid pEA168-CMV-aaRS-4xU6tRNA. A PCR fragment encoding a dominant version of the eukaryotic release factor 1 [eRF1(E55D)] amplified from plasmid peRF1-E55D (38) was subsequently inserted into an expression cassette driven by the EF1 promoter into pEA168-CMV-aaRS-4xU6tRNA using KpnI/MluI restriction sites, yielding pNESPylRS-eRF1dn-tRNA.

### Cell culture.

HEK293T (77) and HeLa TZM-bl indicator cells (78) were maintained in Dulbecco’s modified Eagle’s medium (Thermo Fisher Scientific) supplemented with 100 U/mL penicillin, 100 μg/mL streptomycin (PAN Biotech), and 10% fetal calf serum (FCS; Sigma-Aldrich). Both cell lines were regularly monitored for mycoplasma contamination using the MycoAlert mycoplasma detection kit (Lonza Rockland). Primary CD4^+^ T cells were cultured in RPMI 1640 containing l-glutamine supplemented with 100 U/mL penicillin, 100 μg/mL streptomycin (PAN Biotech), 10% heat-inactivated FCS, and 5% human AB serum (Sigma-Aldrich).

### Isolation of primary cells.

Primary human CD4^+^ T cells were isolated from buffy coats obtained from healthy and anonymous blood donors at the Heidelberg University Hospital Blood Bank following the regulations of the local ethics committee. CD4^+^ T cells were isolated using EasySep Direct Human T Cell isolation kit (Stemcell Technologies) according to the manufacturer’s instructions and activated by incubation in the presence of 100 U/mL IL-2 (Sigma-Aldrich) and T Cell TransAct human (Miltenyi Biotec) for 72 h.

### Virus particle production.

HEK293T cells were seeded in T175 tissue culture flasks the day before (~15 MIO cells) or a 6-well plate (4 × 10^5^ cells/well) and transfected using calcium phosphate precipitation according to standard procedures (~80% confluence). Cells were cotransfected with a 50-μg/flask or 3-μg/well total DNA of pNLC4-3* (HIV-1*) or pNLC4-3*CA14^TAG^ (HIV-1*CA14^TAG^) and plasmid pNESPylRS-eRF1dn-tRNA in a molar ratio of 2.22:1. At 6 h p.t., medium was removed, and fresh complete DMEM containing a final concentration of 500 μM CpK (SiChem; stock solution of 100 mM was prediluted 1:4 in 1 M HEPES shortly before use), and 100 μM ascorbic acid (Sigma-Aldrich; stock solution 10 mM) was added. At 48 h p.t., the tissue culture supernatant was harvested and filtered through 0.45 μm nitrocellulose filters. For labeling the CA protein, 250 nM Tetrazine-SiR (Spirochrome; stock solution 1 mM) was added to the filtered supernatant, and samples were incubated at 37°C for 30 min. Particles were then concentrated by ultracentrifugation through a 20% (wt/vol) sucrose cushion at 28,000 rpm using a Beckman TLA-100 fixed angle-rotor (Beckman Coulter) for 90 min or for the 6-well format at 44,000 rpm using a Beckman TLA-55 fixed angle-rotor (Beckman Coulter) for 45 min at 4°C. Pellets were gently resuspended in phosphate-buffered saline (PBS) containing 10% FCS and 10 mM HEPES (pH 7.5) and stored in 5-μL aliquots at −80°C.

### Immunoblotting and in-gel fluorescence.

Virus samples were mixed 1:10 with SDS sample buffer (150 mM Tris-HCl, pH 6.8, 6% [wt/vol] SDS, 30% glycerin, 0.06% bromophenol blue, 20% β-mercaptoethanol) and boiled at 95°C for 15 min. Ten microliters of HIV-1* and 40 μL HIV-1*CA^SiR^ lysates were subjected to SDS-PAGE (15%; acrylamide:bisacrylamide, 200:1). Cell lysates were generated from transfected HEK293T cells. At 40 h p.t., cells were washed with PBS, trypsinized, and resuspended in PBS. One milliliter of cell suspension was mixed with 300 μL SDS sample buffer and boiled at 95°C for 15 min. Ten microliters cell lysate was subjected to SDS-PAGE. Proteins were transferred to a nitrocellulose membrane (Millipore) by semidry blotting for 1 h at 0.8 mA/cm^2^. Antigens were stained with the indicated antisera in PBS/0.5% bovine serum albumin (BSA) (sheep αCA, polyclonal, 1:5,000 [in-house]; rabbit αMA, polyclonal, 1:1,000 [in-house]; or rabbit αRT, polyclonal, 1:1,000 [in-house]), followed by staining with corresponding secondary antibodies IRDye in PBS/0.5% BSA (anti-sheep 680CW, 1:10,000 [Rockland]; or anti-rabbit 800CW, 1:10,000 [Li-COR Biosciences]). Detection was performed using a Li-COR Odyssey CLx infrared scanner (Li-COR Biosciences) according to manufacturer’s instructions. CA quantification was performed with ImageStudio LITE software (Li-COR Biosciences) via intensity measurements of CA bands and a serial dilution of recombinant purified CA standard (2.5 ng/μL; in-house) on the same membrane. For in-gel fluorescence, the acrylamide gels were directly scanned using a Li-COR Odyssey CLx infrared scanner (Li-COR Biosciences) set at an emission wavelength of 700 nm.

### Infectivity assays.

Virus amounts were quantified via SYBR Green-based product enhanced reverse transcription assay (SG-PERT [79]). To determine relative infectivity of HIV-1* particles in a luciferase assay, 5,000 TZM-bl cells were seeded in 90 μL DMEM in a 96-well plate. On the following day, infection was performed 10 μL of serial 9-fold dilution of supernatant from virus-producing HEK293T cells transfected with pNLC4-3 or pNLC4-3*. At 48 h p.i., cells were lysed by adding 100 μL Steady-Glo luciferase reagent (Promega). After 10 min, 80 μL of the lysed cell solution was transferred into a white 96-well plate and an Infinite 200 PRO plate reader (TECAN) was used to measure luminescence. Uninfected cells were used as a negative control.

To determine the effect of incorporating CpK and Tet-SiR labeling on virus infectivity, HIV-1* and HIV-1*CA14^SiR^ viral particles (normalized by RT activity) were titrated on TZM-bl cells seeded in 15-well ibidi μ-Slide angiogenesis dishes. At 6 h p.i. 50 μM T-20 (Enfuvirtide; Roche; stock solution 20 mM) was added to prevent second-round infection. Infection rates were scored at 48 h p.i. For this, cells were fixed in 4% paraformaldehyde (PFA; Electron Microscopy Sciences; stock solution 16%) for 15 min, followed by a 20-min incubation in PBS/0.5% (vol/vol) Triton X-100 at room temperature. Immunostaining was performed using an in-house polyclonal rabbit antiserum raised against recombinant HIV-1 MA (1:1,000; in-house) in PBS/0.5% BSA for 1 h at room temperature. Secondary antibody Alexa Fluor 488 donkey anti-rabbit (1:1,000; Thermo Fisher Scientific) in PBS/0.5% BSA was added for 45 min at room temperature. Samples were imaged by SDCM. The mean intensity of the 488 channel (MA IF) was quantified in the noninfected samples imaged in parallel and subtracted as background in each image. The proportion of IF-positive cells was counted in 12 randomly selected fields of view using Fiji (80). To determine the infectivity of virus particle preparations, the number of infected cells per well was calculated by multiplying the percentage of infected cells detected with the number of cells per well (double of seeded cell number the day before). Division by the volume of virus suspension used for infection yielded the number of infectious units (IU)/mL.

### Fixation and immunofluorescence staining of infected cells.

TZM-bl cells (3.33 × 10^3^) were seeded into 15-well μ-Slides angiogenesis dishes (ibidi; cat. no. 81507) the day before infection. Infection at 37°C was performed with an MOI ~0.8 for 6, 9, 12, or 18 h. Subsequently, cells were incubated for 1 h with 15 μM PF74 (Sigma-Aldrich; stock solution, 10 mM in DMSO) in DMEM to allow for efficient detection of nuclear CA by IF (17). Samples were washed with PBS, fixed in 4% PFA for 15 min, permeabilized with PBS/0.5% (vol/vol) Triton X-100 for 20 min, and washed again with PBS. Cells were extracted using ice-cold 100% methanol for 10 min. Afterward, samples were blocked with PBS/2.5% BSA for 15 min, followed by incubation with primary antibodies (rabbit αCA, polyclonal, 1:1000 [in-house]; rabbit αCPSF6, polyclonal 1: [Atlas Antibodies; cat. no. HPA039973]; mouse αhLamin A/C, monoclonal, 1:100 [Santa Cruz Biotech.; cat. no. sc-7292]; mouse αhLaminB1, monoclonal, 1:100 [Santa Cruz Biotech.; cat. no. sc-365962] in PBS/0.5% BSA for 1 h at room temperature. After being washed three times with PBS, secondary antibodies (Alexa Fluor 405, 488, 568, goat/donkey, polyclonal, 1:1,000; Thermo Fisher Scientific) diluted in PBS/0.5% BSA were added for 45 min at room temperature. Samples were washed and stored in PBS at 4°C. For infection of primary CD4^+^ T cells, 20,000 cells were infected with HIV-1* or HIV-1*CA14^SiR^ in a 96-well v-bottom microplate (Greiner Bio-one; cat. no. 650161) in a volume of 40 μL RPMI and transferred at 22 h p.i. onto a PEI-coated 15-well μ-Slide angiogenesis dishes (ibidi). Cells were allowed to adhere for 1 h at 37°C, and PF74 diluted in fresh growth medium was added to a final concentration of 15 μM. Extraction, fixation, and immunostaining were performed after 1 h at 37°C as described above. For the detection of endosome-associated particles, 2 μM mCLING ATTO488 (Synaptic Systems; stock, 50 μM) was added to TZM-bl cells seeded in 15-well μ-Slide angiogenesis and incubated at 16°C for 30 min. Subsequently, the fluorescent probe was removed, HIV-1*CA14^SiR^ particles were added in fresh growth medium, and cells were incubated for an additional 3 h at 37°C (MOI, ~0.8). Cells were fixed for 90 min at room temperature in 4% PFA and 0.2% glutaraldehyde to ensure retention of mCLING at cellular membranes. Nuclei were stained with 5 μg/mL Hoechst (Merck) in PBS for 30 min.

### Cell viability assay.

To test the effect of mCLING ATTO488 (Synaptic Systems) staining on cell viability, TZM-bl cells were seeded into a 96-well plate (5 × 10^3^ cells/well; flat bottom Greiner Bio-one) the day before and incubated in medium supplemented with the indicated concentration of mCLING ATTO488 for 30 min at 16°C. After staining, cells were trypsinized, stained with Trypan blue using standard procedures and analyzed with a TC20 Automated Cell Counter (Bio-Rad).

### Labeling efficiency of immobilized particles.

Fifteen-well μ-Slide angiogenesis dishes (ibidi) were coated with 30 μL/well polyethyleneimine (PEI; 1 mg/mL) for 30 min at room temperature and washed with PBS. Prelabeled HIV-1* and HIV-1*CA14^SiR^ particles were incubated in PBS on PEI-coated microscopy slides for 1 h at 37°C. Subsequently, samples were washed with PBS, fixed in 4% PFA for 15 min and permeabilized with PBS/0.05% (vol/vol) Triton X-100 for 20 min at room temperature. Immobilized particles were blocked with PBS/2.5% BSA for 15 min, and polyclonal rabbit antiserum raised against recombinant HIV-1 CA protein (in-house) was added (1:1,000 in PBS/0.5% BSA for 1 h at room temperature). After being washed three times with PBS, secondary antibody Alexa Fluor 488 donkey anti-rabbit (Thermo Fisher Scientific) at 1:1,000 in PBS/0.5% BSA was added for 45 min at room temperature. Samples were washed and stored in PBS at 4°C.

### Confocal microscopy.

Multichannel z-series with a z-spacing of 200 nm, spanning the whole cell volume (3D), were acquired using a PerkinElmer Ultra VIEW VoX 3D spinning disk confocal microscope (SDCM; Perkin Elmer). A 60× oil immersion objective (numeric aperture [NA], 1.49; Perkin Elmer) was used for imaging of TZM-bl cells or 100× oil immersion objective (NA, 1.49; Perkin Elmer) for primary CD4^+^ T cells and immobilized particles. Images were recorded in the 405-, 488-, 561-, and 640-nm channels.

### STED microscopy.

STED nanoscopy was performed using a λ = 775-nm STED system (Abberior Instruments GmbH) equipped with a 100× oil immersion objective (NA, 1.4; Olympus UPlanSApo). STED images were acquired using the 640-nm excitation laser lines while the 488 and 590 laser line was acquired in confocal mode only. Nominal STED laser power was set to 20% of the maximal power (1,250 mW) with pixel dwell time of 10-μs and 15-nm pixel size. STED images were deconvolved using the software Imspector (Abberior Instruments GmbH) and Huygens Professional Deconvolution (Scientific Volume Imaging).

### Electron microscopy.

HEK293T cells (4 × 10^5^) were seeded in a glass coverslip-bottom petri dish (MatTek, MA, USA), cultured for 16 h at 37°C and then cotransfected with pNLC4-3*CA14^TAG^ and pNESPylRS-eRF1dn-tRNA by using calcium phosphate precipitation. At 6 h p.t., medium was removed and fresh complete DMEM containing a final concentration of 500 μM CpK (SiChem; stock solution, 100 mM was prediluted 1:4 in 1 M HEPES shortly before use), and 100 μM ascorbic acid (Sigma-Aldrich; stock solution, 10 mM) was added. At 44 h p.t., cells were fixed with prewarmed 2% formaldehyde + 2.5% glutaraldehyde in 0.1 M cacodylate buffer (pH 7.4) for 1.5 h at room temperature, then washed in 0.1 M cacodylate buffer and postfixed with 2% osmium tetroxide (Electron Microscopy Sciences) for a 1 h on ice. Cells were subsequently dehydrated through an increasing cold ethanol series (30, 50, 70, 80, 90, and 100%; on ice) and two anhydrous acetone series (at room temperature). The coverslip with cells was then removed from the dish, and cells were flat embedded in Epon resin. Seventy-nanometer-thin sections were cut with an ultramicrotome (Leica EM UC6), collected on Formvar-coated 100-mesh copper EM grids (Electron Microscopy Sciences) and stained with a 3% uranyl acetate in 70% MetOH (10 min) and lead citrate (7 min). Cells sections were observed with a JEOL JEM-1400 electron microscope operating at 80 kV (Jeol Ltd.), equipped with a bottom-mounted 4K by 4K pixel digital camera (TemCam F416; TVIPS GmbH).

### CLEM and electron tomography.

SupT1 cells were distributed in a 96-well plate (2 × 10^5^ cells/well; U-bottom; Greiner Bio-one, 650180) and preincubated for 16 h with 1 μm aphidicolin (APC; Merck). Cells were pelleted (200 × *g*, 3 min) and resuspended in complete RPMI medium containing HIV-1*CA14^SIR^ particles (MOI, ~0.4). Cells were incubated with viral particles for 120 min at 16°C to adsorb the virus and synchronize virus entry. Samples were then processed for CLEM and ET as described previously (16). In brief, cells were transferred to a glass-bottomed microwell of a MatTek dish (MatTek) containing carbon-coated and retronectin-coated sapphire discs (Engineering Office M. Wohlwend). Samples were high pressure frozen, and sapphire discs were then transferred from liquid nitrogen to the freeze-substitution (FS) medium (0.1% uranyl acetate, 2.3% methanol, and 2% H_2_O in acetone) tempered at −90°C. Samples were FS-processed and embedded in Lowicryl HM20 resin (Polysciences) according to a modified protocol of Kukulski et al. (81). For CLEM-ET, thick resin sections (250 nm) were cut and placed on a slot (1 × 2 mm) EM copper grids covered with a Formvar film (Electron Microscopy Sciences; FF2010-Cu). Grids were decorated with fiducial marker and stained with Hoechst to visualize nuclear regions. Light microscopy Z stacks of sections were acquired by PerkinElmer UltraVIEW VoX 3D Spinning-disc Confocal Microscope (Perkin Elmer) using a 100 × oil immersion objective (NA 1.49; Nikon), with a *z-*spacing of 200 nm and excitation with the 405-, 488-, 561-, and 633-nm laser line. Acquired z stacks were visually examined using Fiji software (80), and intracellular CA(SiR)-positive signals were identified. EM grids were decorated with 15 nm protein-A gold particles for tomogram alignment and stained with uranyl acetate and lead citrate. Grids were loaded to a Tecnai TF20 (FEI) electron microscope (operated at 200 kV) equipped with a field emission gun and a 4K by 4K pixel Eagle CCD camera (FEI). Positions of CA(SiR) signals were precorrelated with imported SDCM images in SerialEM as described previously (82). Single-axis electron tomograms were carried out. Tomographic tilt ranges were typically from −60° to 60° with an angular increment of 1°. The pixel size was 1.13 nm. Alignments and 3D reconstructions of tomograms were done with IMOD software (83). Postcorrelation was performed using eC-CLEM plugin in Icy software (84).

### Image analysis.

Microscopy images were screened and filtered in Fiji/ImageJ (80) with a mean filter and background subtraction. Infected cells were quantified in Fiji via segmentation and counting of nuclei and the cell counter to manually quantify the number of positive cells. To determine labeling efficiency of click-labeled particles, CA(SiR) intensities of detected immobilized particles based on CA(IF) were quantified using the spot detector of the software Icy (84). Five ROIs without particles were measured, and mean intensity in the SiR channel was subtracted as background. The threshold was set to *t* = 1,000 AU CA(IF) detected spots with intensities above the threshold were classified as CA(SiR) positive.

To analyze particle distribution and intensity measurements throughout the volume of cells, z-image series were reconstructed in 3D space using Imaris 9.2 software (Bitplane AG). Individual HIV-1 CA(IF) objects were automatically detected using the spot detector Imaris module, which created for each fluorescent signal a 3D ellipsoid object with 300-nm estimated diameter in *x-y* dimensions and 600 nm in *z*. The local background of each individual spot was subtracted automatically. Subsequently, the mean signal intensity in the CA(SiR) channel was quantitated within all objects. The threshold for SiR intensity was set to *t* = 7,000 AU and adjusted manually for each image by visual inspection. Spots detected in SiR-clusters were excluded. Nuclear objects were manually identified based on the lamin A/C staining. NE-associated objects were classified based on lamin A/C intensities. Every image was manually inspected and a threshold for NE-associated objects was set in the range of 6,300 to 9,100 AU. All other particles were classified as PM/cytoplasm (in the cytoplasm/at plasma membrane).

To identify postfusion cores by mCLING ATTO488 staining, CA SiR-positive objects were automatically detected using the spot detector Imaris module and the mCLING ATTO488 mean signal intensity colocalizing with each object was quantitated. Particles located outside of the cell, attached to the plasma membrane, or within clusters of foci were excluded from the analysis. Detected objects with the lowest mCLING ATTO488 intensities were individually inspected. Only particles showing a distinct CA SiR focus lacking the mCLING ATTO488 signal were classified as mCLING negative.

Fiji standard “greyscale” lookup table (LUT) was used to visualize single channel images and “Fire” for single channel STED images.

### Data visualization and statistical analysis.

Statistical significance was assessed using Prism v9.1.0 (GraphPad Software Inc). A two-tailed nonpaired Mann-Whitney test (α = 0.05) was used to assess the statistical significance of nonparametric data. Data were plotted using Prism v9.1.0 or the Python statistical data visualization package seaborn v.0.10.0 (Waskom 2020). Graphs show mean/median with error bars as defined in the figure legends.

### Data availability.

The data supporting the findings of this study are available in the article and supplement material. All primers and constructs used in this study are available upon request. For original data, please contact corresponding author.
