# Supplementary figures and images for: Direct Capsid Labeling of Infectious HIV-1 by Genetic Code Expansion Allows Detection of Largely Complete Nuclear Capsids and Suggests Nuclear Entry of HIV-1 Complexes via Common Routes
Source: mBio. 2022 Aug 16;13(5):e01959-22. doi: 10.1128/mbio.01959-22 (PMC9600849; doi:10.1128/mbio.01959-22)

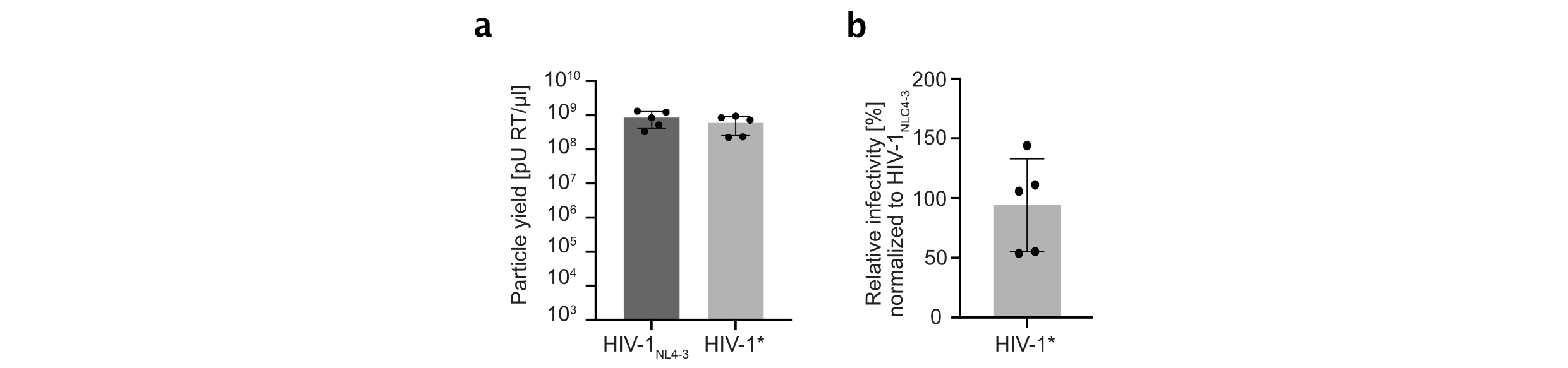

Supplement: FIG S1 [file mbio.01959-22-s0001.tif]

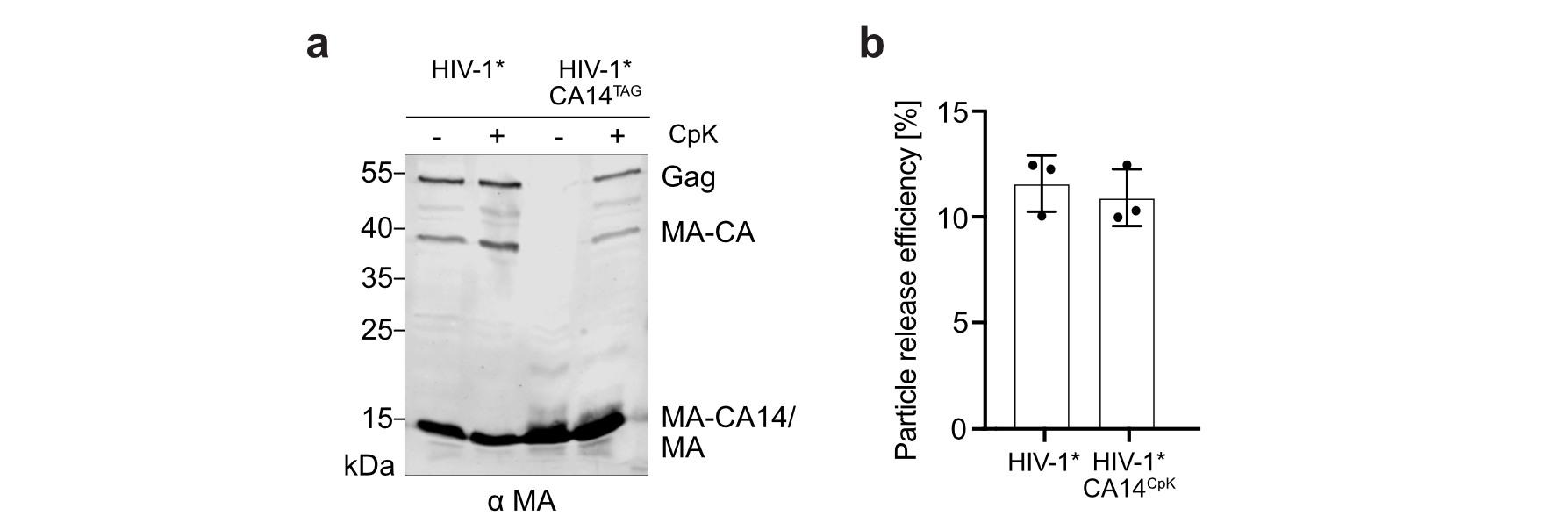

Supplement: FIG S2 [file mbio.01959-22-s0002.tif]

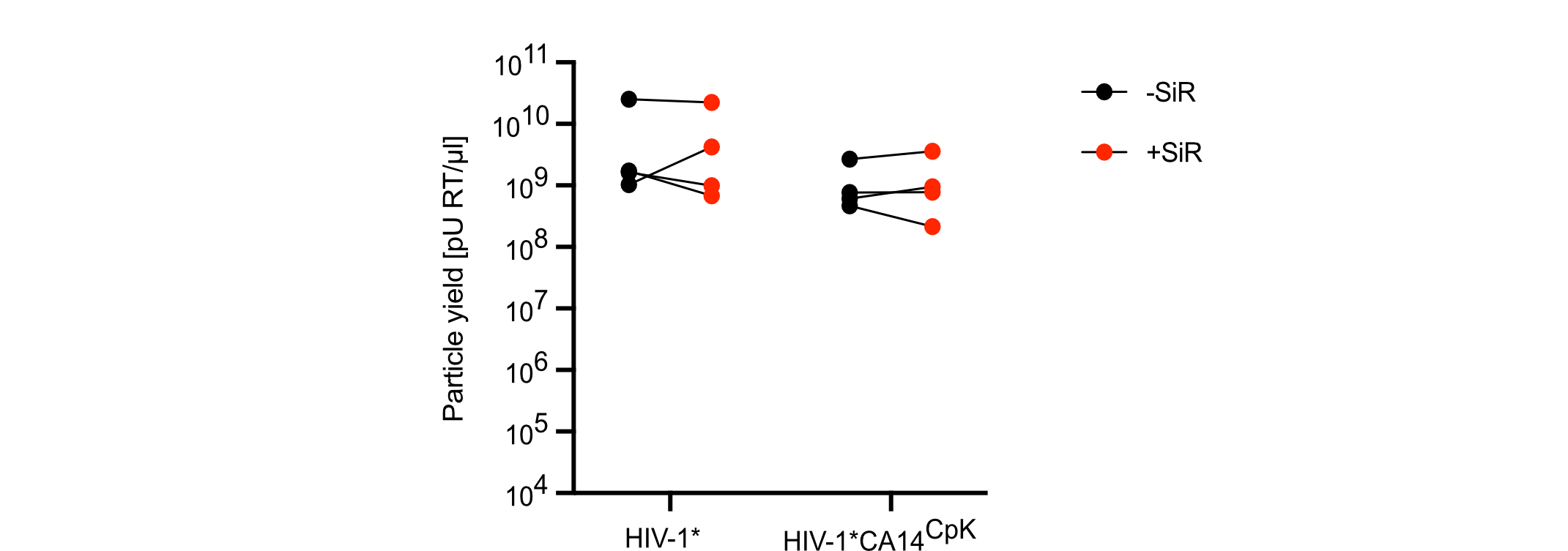

Supplement: FIG S3 [file mbio.01959-22-s0003.tif]

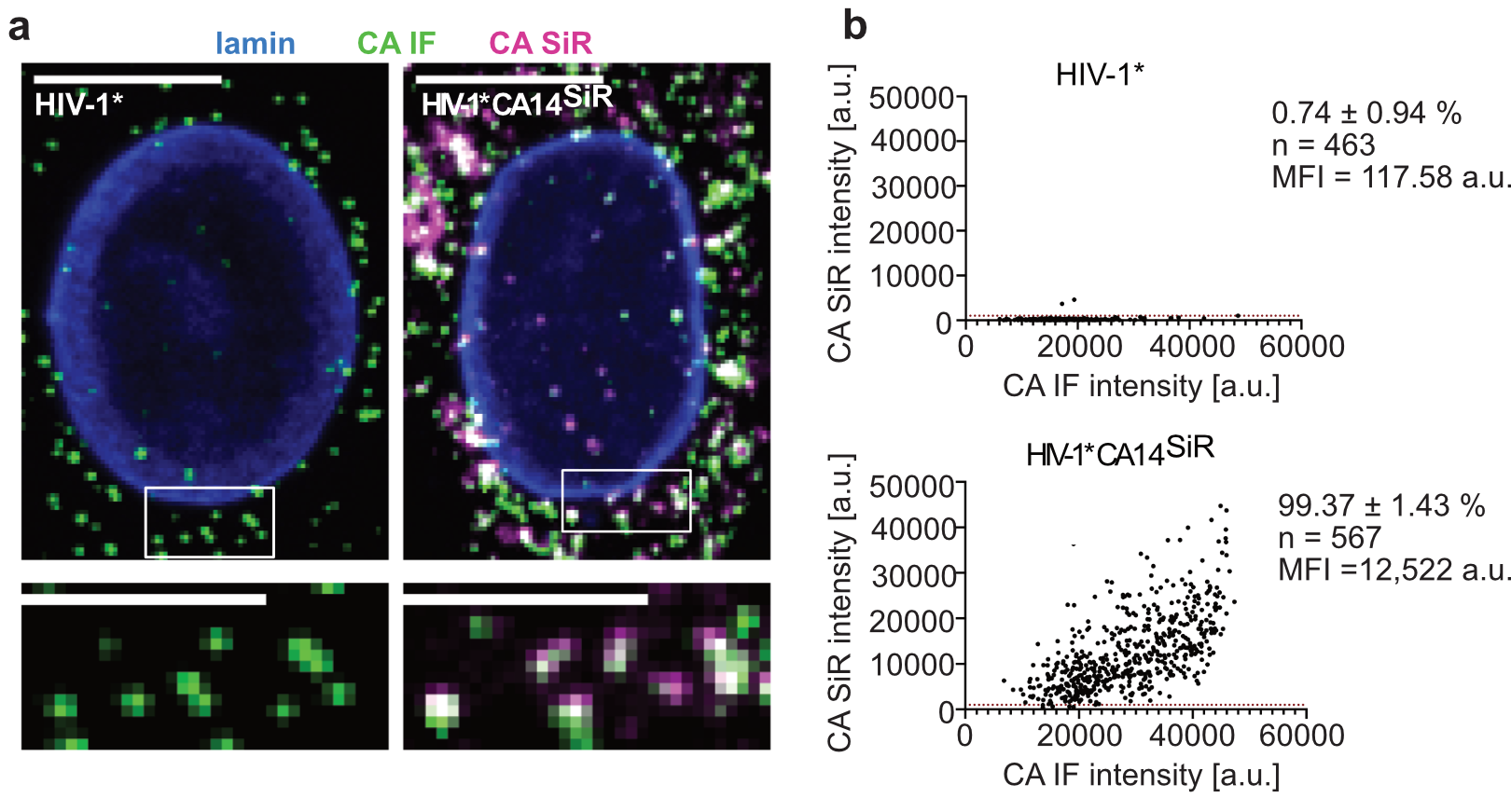

Supplement: FIG S4 [file mbio.01959-22-s0004.tif]

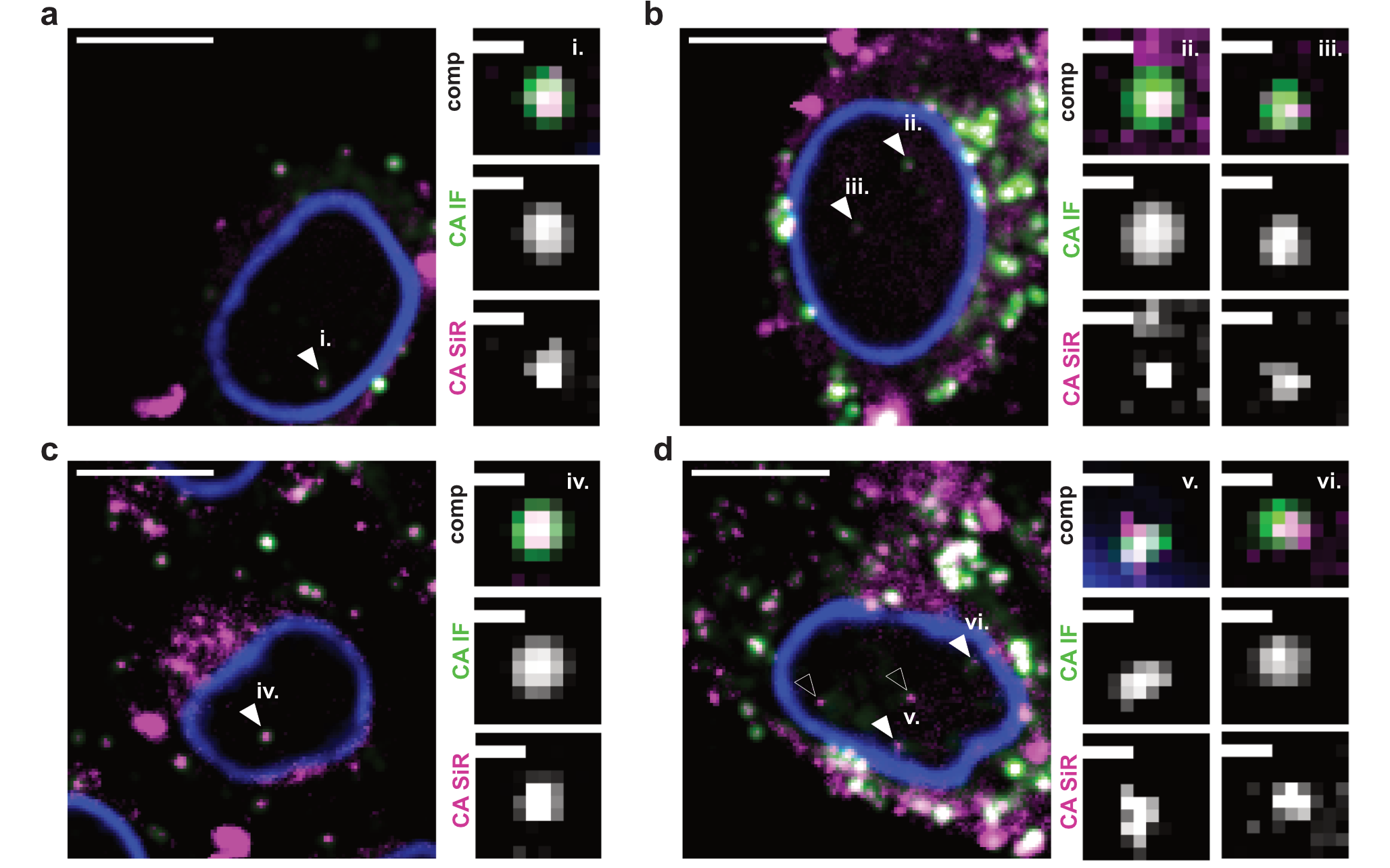

Supplement: FIG S5 [file mbio.01959-22-s0005.tif]

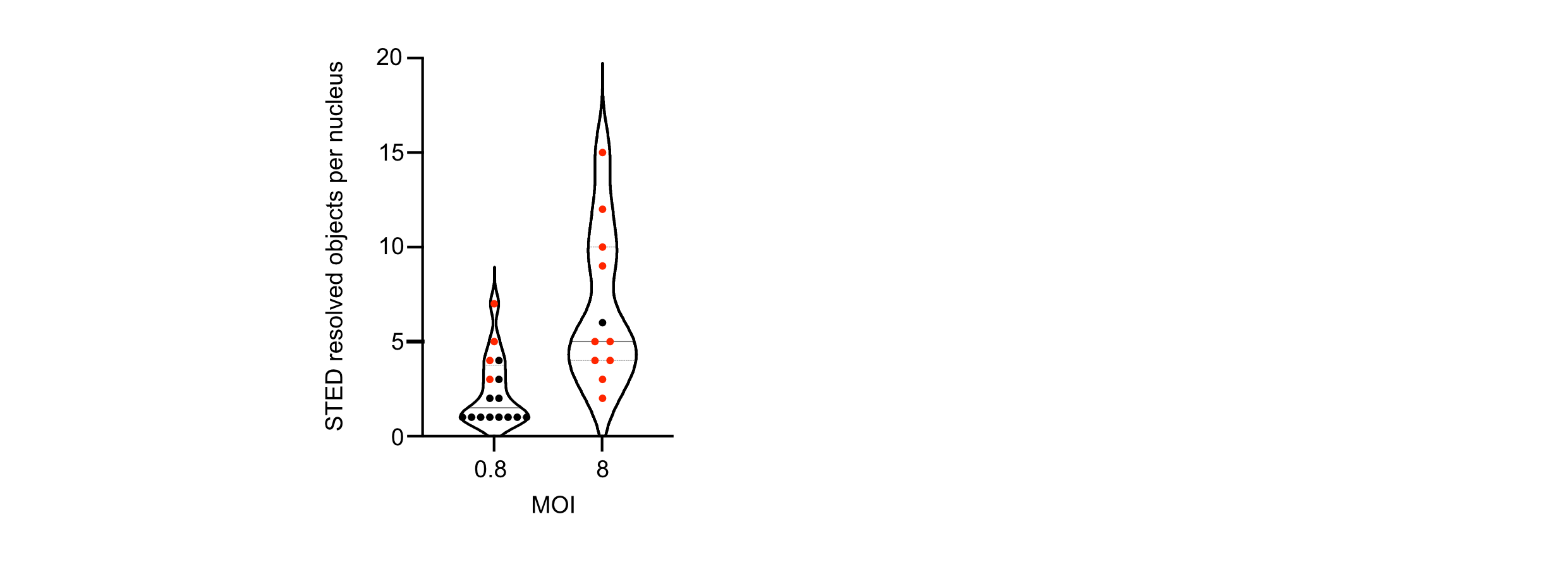

Supplement: FIG S6 [file mbio.01959-22-s0006.tif]

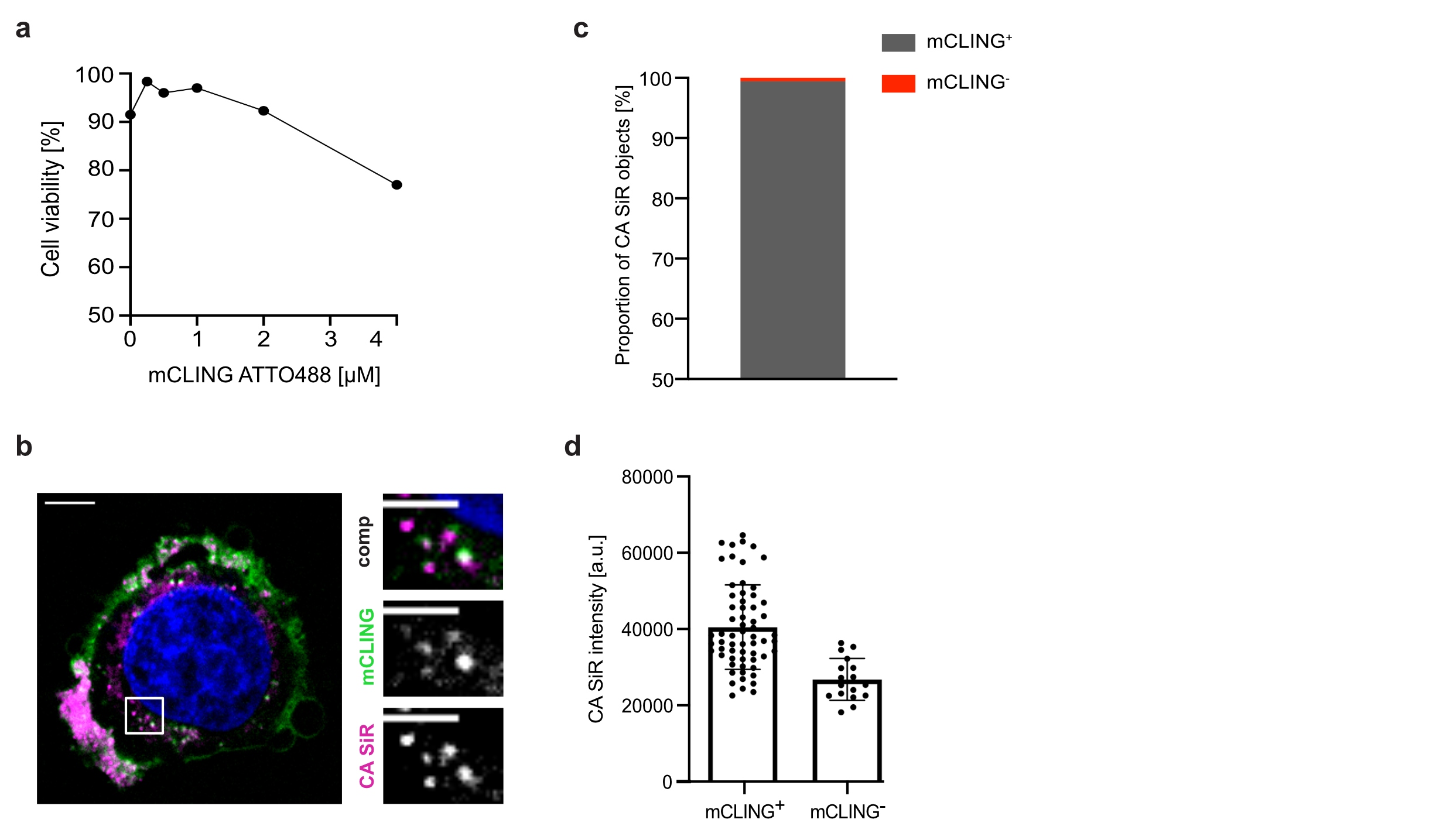

Supplement: FIG S7 [file mbio.01959-22-s0007.tif]

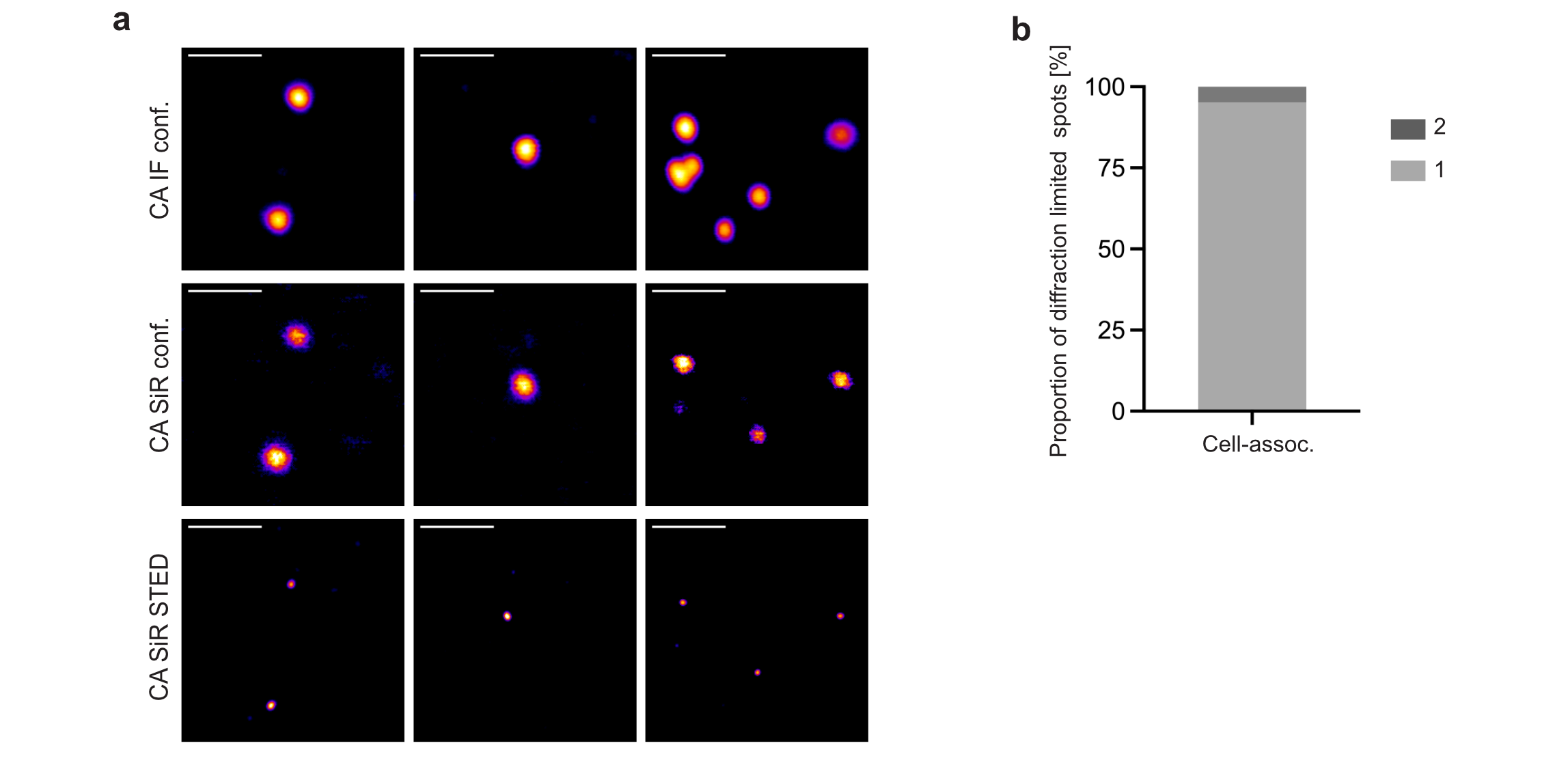

Supplement: FIG S8 [file mbio.01959-22-s0008.tif]

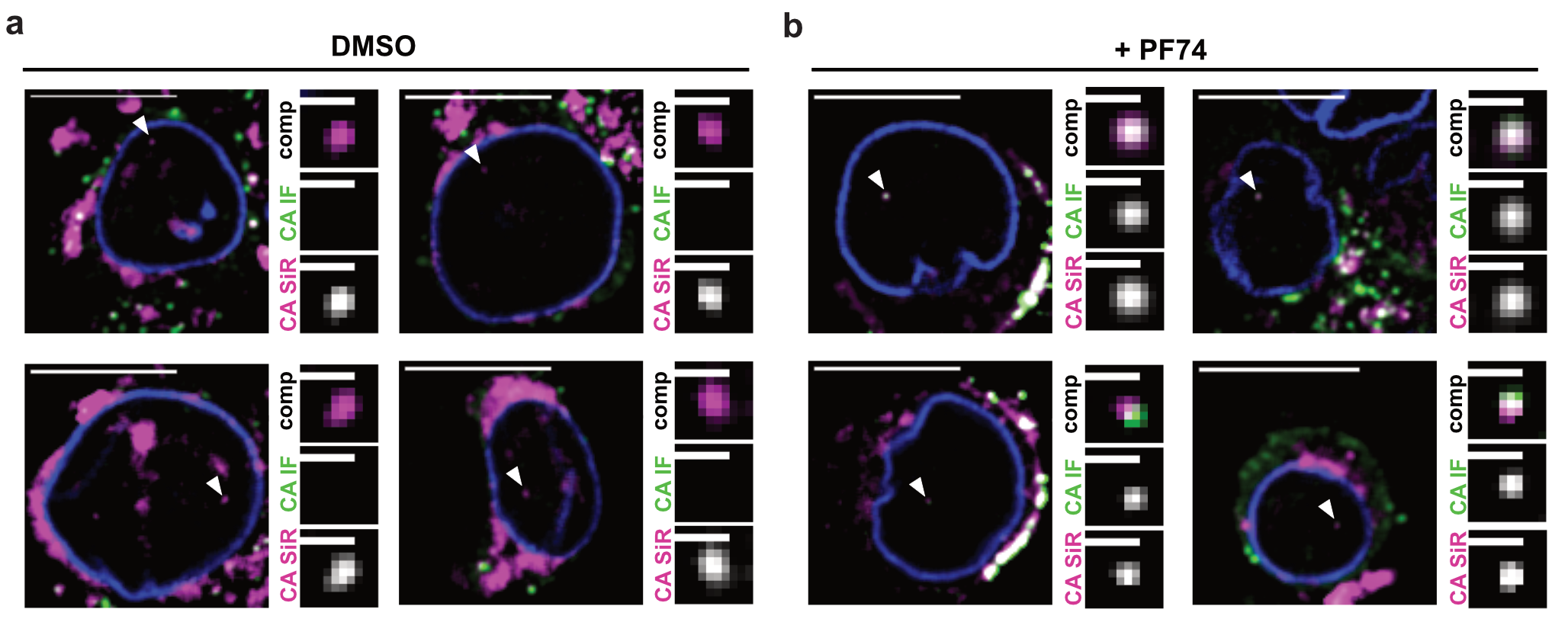

Supplement: FIG S9 [file mbio.01959-22-s0009.tif]
